# The mutational landscape of a US Midwestern breast cancer cohort reveals subtype-specific cancer drivers and prognostic markers

**DOI:** 10.1186/s40246-023-00511-6

**Published:** 2023-07-15

**Authors:** Neetha Nanoth Vellichirammal, Yuan-De Tan, Peng Xiao, James Eudy, Oleg Shats, David Kelly, Michelle Desler, Kenneth Cowan, Chittibabu Guda

**Affiliations:** 1 Department of Genetics, Cell Biology and Anatomy, University of Nebraska Medical Center, Omaha, NE 68198 USA; 2 Eppley Institute for Research in Cancer and Allied Diseases, University of Nebraska Medical Center, Omaha, NE USA; 3 Center for Biomedical Informatics Research and Innovation, University of Nebraska Medical Center, Omaha, NE 68198 USA; 4 Fred and Pamela Buffett Cancer Center, University of Nebraska Medical Center, Omaha, USA

## Abstract

**Background:**

Female breast cancer remains the second leading cause of cancer-related death in the USA. The heterogeneity in the tumor morphology across the cohort and within patients can lead to unpredictable therapy resistance, metastasis, and clinical outcome. Hence, supplementing classic pathological markers with intrinsic tumor molecular markers can help identify novel molecular subtypes and the discovery of actionable biomarkers.

**Methods:**

We conducted a large multi-institutional genomic analysis of paired normal and tumor samples from breast cancer patients to profile the complex genomic architecture of breast tumors. Long-term patient follow-up, therapeutic regimens, and treatment response for this cohort are documented using the Breast Cancer Collaborative Registry. The majority of the patients in this study were at tumor stage 1 (51.4%) and stage 2 (36.3%) at the time of diagnosis. Whole-exome sequencing data from 554 patients were used for mutational profiling and identifying cancer drivers.

**Results:**

We identified 54 tumors having at least 1000 mutations and 185 tumors with less than 100 mutations. Tumor mutational burden varied across the classified subtypes, and the top ten mutated genes include *MUC4, MUC16, PIK3CA, TTN, TP53, NBPF10, NBPF1, CDC27, AHNAK2*, and *MUC2*. Patients were classified based on seven biological and tumor-specific parameters, including grade, stage, hormone receptor status, histological subtype, Ki67 expression, lymph node status, race, and mutational profiles compared across different subtypes. Mutual exclusion of mutations in *PIK3CA* and *TP53* was pronounced across different tumor grades. Cancer drivers specific to each subtype include *TP53, PIK3CA, CDC27, CDH1, STK39, CBFB, MAP3K1*, and *GATA3*, and mutations associated with patient survival were identified in our cohort.

**Conclusions:**

This extensive study has revealed tumor burden, driver genes, co-occurrence, mutual exclusivity, and survival effects of mutations on a US Midwestern breast cancer cohort, paving the way for developing personalized therapeutic strategies.

**Supplementary Information:**

The online version contains supplementary material available at 10.1186/s40246-023-00511-6.

## Introduction

Female breast cancer is one of the most commonly diagnosed cancers globally, with an estimated 2.26 million in 2020, and the second most frequent cause of death related to cancer [1, 2]. Breast cancer is a heterogeneous disease with a high degree of variability in tumor morphology both across the cohort and within a patient resulting in unpredictable therapy resistance, metastasis, and clinical outcome [3–5]. Recent statistics indicate that despite remarkable advances in early diagnostic methods and clinical management of the disease, breast cancer still contributes to 7.2% of all cancer deaths [6]. Breast tumor stratification based on histological grade, size, lymph node status, stage, and hormone receptor status currently serves to provide prognostic predictors and guides clinical decision making. In addition to these factors, integrating molecular-based classification using gene panels and whole genome or transcriptome sequencing technology can reveal previously unseen biological properties of the tumor, impacting clinical management [7–9]. This study attempts to characterize the molecular features of breast cancer in a predominantly rural midwestern population.

Several studies focused on the molecular profiles of breast tumors have demonstrated that intrinsic molecular characteristics of the tumor correlate with survival outcomes or treatment responses [10–12]. Investigating the complex genomic landscape of this heterogeneous tumor has also led to the identification of novel molecular subtypes and the discovery of actionable biomarkers [6, 13–15]. For example, a comprehensive global gene expression analysis identified a novel breast cancer subtype, ‘Claudin-low,’ characterized by low expression of Claudin genes and enrichment of cell adhesion proteins [16, 17]. The Claudin-low subtype was prevalent in triple-negative breast cancer, and this identification of a new subtype emphasizes the importance of supplementing classic pathological markers with molecular markers. Along with gene expression profiles, comprehensive mutational profiling of breast tumors can identify driver genes specific to subtypes [18, 19]. In addition, these genomic characterizations have also led to the development of widely used commercial multigene prognostic signatures like PAM50, MammaPrint, and OncotypeDX that predict chemotherapy sensitivities and metastasis risk. Even though these genomic assays provide a definitive predictive advantage, they are limited to a subset of patients with intermediate prognosis risk based on their grade, Ki67 expression profile, or hormone receptor status [20, 21]. In addition, triple-negative breast cancers with high heterogeneity in prognosis warrant better molecular prognostic signatures. Moreover, identifying actionable mutations in these aggressive breast cancer subtypes can aid in the development of tailored therapy for a better prognosis. Therefore, additional studies are warranted to characterize the molecular profile of individual breast tumors and integrate this information with clinicopathological features to improve current prediction tools.

To better understand the complex genomic architecture of breast tumors, we conducted a large multi-institutional genomic analysis of normal and tumor samples from breast cancer patients. The Fred and Pamela Buffett Cancer Center (FPBCC) and its affiliated hospital network have been maintaining a unique resource, the Breast Cancer Collaborative Registry (BCCR) [22], which is a part of the integrated Cancer Repository for Cancer Research (iCaRe2, https://icare2.unmc.edu). BCCR catalogs longitudinal data on BC patients that include several clinicopathological parameters such as tumor stage, grade, hormone receptor status, and histological subtype, in addition to long-term patient follow-up, therapeutic regimens, and treatment response. The uniqueness of our patient cohort includes well-documented clinical and treatment history of patients with germline-matched high-quality whole-exome sequencing data that can be analyzed on the basis of each clinicopathological parameter. In this study, we characterized the tumor mutational profiles of the BCCR cohort based on seven different criteria and investigated potential clinical relevance associated with their mutational profiles. This unique study uses well-annotated and curated breast cancer patient data from seven institutions in three US Midwestern states (Nebraska, North Dakota, and South Dakota) and integrates clinical and genomic information to characterize and identify potential therapeutic targets for precision medicine. Our study revealed significant findings, such as the prominent mutual exclusion of mutations in *PIK3CA* and *TP53* across various tumor grades. Additionally, we identified specific cancer drivers for each subtype, including *TP53, PIK3CA, CDC27, CDH1, STK39, CBFB, MAP3K1*, and *GATA3*.

## Results

### Overview of the breast cancer cohort in this study

Clinicopathological features of the 554 breast cancer patients enrolled in this study are summarized in Table 1. The median age at diagnosis was 57.5 years, and the majority of the breast cancer patients in this study (90%) were Caucasians. Male breast cancer accounted for 2.2% of the patients. Three patients in our study group had bilateral tumors–tumor samples with the worst T stage were included for those patients. Most of the patients had ER or PR+/Her2-ve receptor expressions (66.1%); 15.3% had triple-negative subtypes. Patients with high Ki67-high expression were prevalent (61%). After a median follow-up of 7.6 years, approximately 16% of our cohort experienced relapse at some point, and 14% had distant metastasis. The most common sites of metastasis were visceral, bone and skin.

**Table 1:** Patient characteristics

Total number of patients	554
Median follow-up time	7.4 Years
Median age at diagnosis (range)	57.5 Years (22–85)
	≥ 50 years = 402
	< 50 years = 152
Race/ethnicity	White = 500, Black = 29 Hispanic (White or Other) = 14, Asian = 6 Multiracial/Other = 10, Unknown = 8
Ki67(%)	Low (≤ 15%) = 104, High (≥ 15%) = 161
Tumor stage at Dx	*T* = 292, *T*2 = 201, *T*3 = 41, *T*4 = 8, Tis = 8
Gender	*F* = 542, *M* = 12
Molecular subtype	TN = 74, ER/PR + ;HER2 + = 69 ER/PR + ;HER2- = 362, ER/PR-;HER2 + = 21 ER/PR + , unknown Her2 status = 4
Nodal status	N0 = 320, N + = 230
Vital status	Alive = 394, Deceased = 116 Unknown/Lost to follow up = 44
Subtype by FISH or IHC	Her2 Type = 12, Luminal = 196 Luminal A = 161, Luminal B = 127 Triple negative = 85
Paired samples (Normal)	Yes = 554
BRCA1-mutation	Yes = 8, no = 546
BRCA2-mutation	Yes = 12, no = 542
Tumor histology	Invasive ductal adenocarcinoma = 473 invasive lobular carcinoma = 52 mixed carcinoma = 5, Other = 2
Menopausal status	Yes = 244, no = 36
Therapy	Any chemotherapy = 390 Neoadjuvant chemotherapy = 75
First site of distant metastasis	Visceral = 14, bone = 15, brain/CNS = 2 Skin, other = 5, multiple = 24

### Mutation profiles of breast tumors

Mapping of sequencing reads to the reference genome showed an average coverage depth of above 100× for normal samples and above 200× for tumor samples in the targeted region. We identified 308,788 somatic mutations in our cohort using the standard mutation prediction pipeline, including missense, non-sense, splice-site mutations, and translation start site mutations. We identified 54 tumors having at least 1000 mutations and 185 tumors with less than 100 mutations in the coding region. The top ten mutated genes in this cohort include *MUC4*, *MUC16*, *PIK3CA, TTN*, *TP53*, *NBPF10*, *NBPF1*, *CDC27*, *AHNAK2*, and *MUC2*. Most of the identified mutations were missense (88%), whereas nonstop mutations and translation start site mutations were less than 0.2% in this cohort.

Patients recruited in this study were subdivided, and mutation profiles were compared across different groups based on demographic and clinical characteristics. Patients were subdivided into different comparison groups based on tumor characteristics, including grade (Grade 1-3), stage (Stages 1, 2, 3 and 4) at the first breast cancer diagnosis, subtype (ER or PR+/Her2-ve, ER or PR+/Her2+ ve, ER- and PR-/Her2-ve, triple-negative), Ki67 staining grade (high, low), histology (invasive lobular carcinoma, invasive ductal carcinoma, others), presence (NPlus) or absence (N0) of lymph node involvement and race (Caucasian, African-American). The mutational profile of each comparison group will be described in detail in the following sections. In each comparison, we present the frequently mutated genes, the percentage of deleterious variants in these genes, Tumor Mutation Burden (TMB), mutational signatures, APOBEC enrichment, co-occurring or mutually exclusive mutations, and mutations associated with survival.

### Comparison of mutation profiles across tumor grade

**Fig. 1:**
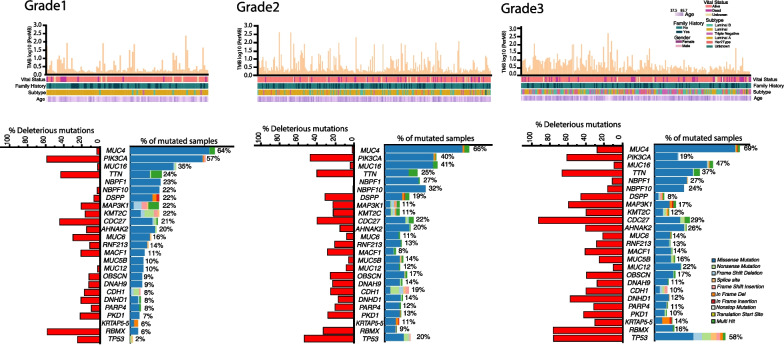
Distribution of patient cohort across the three grades. The upper panel of the figure provides the distribution of tumor mutation burden for each patient assigned to a particular grade. Patient characteristics, including vital status, family history, subtype status, and age, are also included for each group. The bottom panel represents the top mutated genes across the three groups. The percentage of deleterious variants in each gene is also represented, along with the type of mutation detected

Gene-wise and sample-wise comparisons were made across the three grades. Top mutated genes and the deleterious mutations were different across the three tumor grades. *PIK3CA*, *DSPP*, *KMT2C*, and *MAP3K1* were mutated in more than 20% of grade 1 tumors, whereas *TP53*, *MUC12*, and *AHANK2* mutations were prevalent in grade 3 tumors (Fig. 1).

**Fig. 2:**
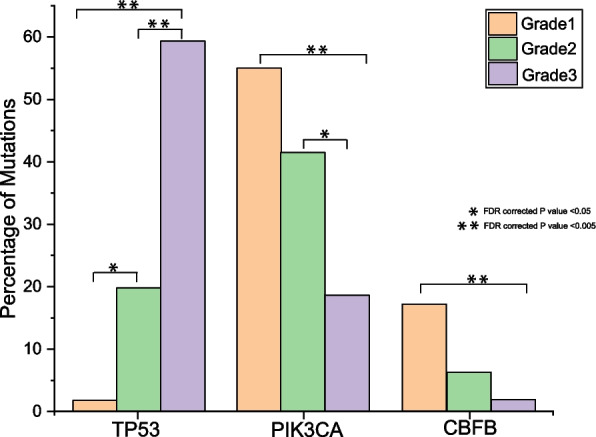
Distribution of differentially mutated genes across grades. The distribution of mutations *TP53, PIK3CA*, and *CBFB,* are shown in the graph. Fisher’s test P-value is provided for statistically significant comparisons

Even though the percentage of samples with *PIK3CA* and *MAP3KI* mutations were relatively small in grade 3, more than 60% of the mutations were deleterious. The percentage of samples with *TP53* mutations was the highest in grade 3 tumors, whereas grade 1 tumors had the highest percentage of *PIK3CA* and *CBFB* mutations (Fig. 2).

Average TMB showed a gradual increase from grade 1 to grade 3 tumors, though not significantly different (Additional file 2: Fig. S1). Genes such as *CDC27, DNHD1*, and *RBMX* had no increase in the number of mutations from grade 1 to 3 but recorded a significant jump in the number of deleterious mutations. Using different statistical tools, we identified variants associated with the etiology of cancer having differential mutation prevalence across tumor grades with *MUC20*, *TP53*, *and RUNX1T1* higher in grade 3 tumors, while *PIK3CA* and *CBFB* were higher in grade 1 tumors (Additional file 1: Table S1). We also noted that a higher percentage of patients in grade 3 had adverse outcomes than other stages when survival status was compared (Chisq *P* value = 0.01).

The study investigated specific subgroups for gene mutations and found that grade 1 tumors had many gene mutations that co-occurred. The top 20 gene sets with co-occurring or mutually exclusive mutations (ZF1). Some gene mutations, such as *MUC16-QRICH2* and *RNF213-TTN*, co-occurred frequently, while *CDC27-NBPF10* mutations were mutually exclusive (Additional file 1: Table S2). A small percentage of grade 1 tumors had an APOBEC-enriched mutagenesis signature, but the mutational load was not significantly different from non-APOBEC-enriched samples. De novo mutational signatures identified in grade 1 tumors included COSMIC signatures 1, 5, 29, and 38 (ZF2). The study used *MutSigCV* to identify genes with significantly higher mutation rates in grade 1 tumors. *PIK3CA, CBFB, CDC27, MAP3K1*, and *ESRRA* were among the genes that were cancer drivers (Additional file 1: Table S3). Patients with *PIK3CA* or *CBFB* mutations had lower survival rates than those without mutations (Additional file 2: Fig. S2A, ZT1).

In grade 2 tumors, there were many co-occurring mutations, including *MUC5B-OBSCN, MUC5B-DNAH9, RNF213-DCHS2, RNF213-AHNAK2*, and *MUC5B-HERC2* (ZF1, Additional file 1: Table S4). *TP53-CDH1, DSPP-CDH1, NBF10-MUC5B, NBF10-MUC16* had mutually exclusive mutations. Grade 2 tumors had a higher percentage of genes with mutational co-occurrence compared to grade 1 tumors. *MutSigCV* analysis identified *TP53, MUC2, PIK3CA, CDH1, CDC27*, and *ERBB2*, as cancer drivers in grade 2 tumors (Additional file 1: Table S5). We identified 4 COSMIC mutational signatures (SBS29, SBS5, SBS38, and SBS2 in grade 2 samples (ZF3). *HERC2, MUC5B, PKD1*, and *AHNAK2* mutations were associated with poor survival in patients with grade 2 tumors (Additional file 2: Fig. S2B, ZT2).

In grade 3 tumors, mutations in *TP53* co-occurred with *RBMX1, OBSCN, DSPP, NBPF1*, and *TTN* mutations (ZF1). *MUC16* mutations also co-occurred with *RBMX, CDC27*, and *AHNAK2*. *MutSigCV* analysis identified *TP53, CDC27, PIK3CA, CDH1, STK39, and FOXO3* as possible cancer drivers in grade 3 tumors (Additional file 1: Table S6). Patients with *TP53* mutations had poorer survival rates compared to wild type *TP53* (Additional file 2: Fig. S2C). Though the samples sizes were extremely small, mutations in *TP53, NBPF1, RBMX, MACF1*, and *CRIPAK* were associated with an increased risk of relapse (ZT3). *CRIPAK* and *NBPF1* mutations conferred higher metastasis risk in grade 3 tumors (ZT3).

APOBEC signature was enriched in 17 grade 3 tumors (9%), similar to grade 2 tumors. Four COSMIC mutational signatures were identified in grade 3 tumors; SBS29, SBS13, SBS26, and SBS38 (ZF4).

### Comparison of mutation profiles across tumor subtypes

**Fig. 3:**
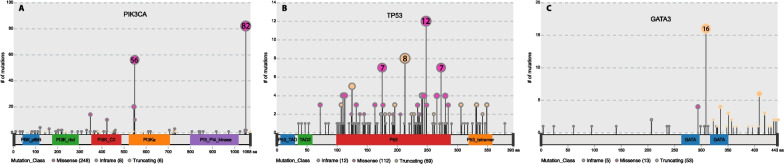
Distribution of mutations across *PIK3CA*, *TP53*, and *GATA3* genes. The number and type of mutations in the different protein domains are represented in the figure

Our patient cohort was categorized into four subtypes based on hormone receptor status. The ER/PR + veHER2-ve subtype was the largest (70%). The triple-negative subtype had significantly higher TMB when compared to all other subtypes, and ER/PR + veHER2-ve had the lowest (Additional file 2: Fig. S3). *PIK3CA* mutations were more frequent in ER/PR + veHER2-ve subgroup, and *MUC16, MUC5B, QRICH2*, and *SVEP1* mutations were frequent in ER/PR + veHER2 + ve subgroup (Fig. 3A, Additional file 2: Fig. S4).

More than 48% of the mutations in *PRAMEF2* were deleterious in this subtype. *MUC4, NBPF1,* and *CSMD3* gene mutations were recurrent in the ER/PR-ve HER2 + ve patients. More than 60% of the mutations identified in *PIK3CA, TTN,* and *ZNF717* in this subtype were deleterious. ER/PR-veHER2 + ve patients had recurrent mutations in *MUC4, NBPF1*, and *CSMD3*, and triple-negative patients had more mutations in *TTN, FLG, SYNE1, LRP1B*, and *PRKDC*, as well as deleterious mutations in *XIRP2*, *SVEP1, OR4A16, DST, CSMD3*, and *FLG*.

Cancer-associated variants differentially mutated across subtypes were identified. Most of these mutations were identified in *HLA-A, HLA-DRB1, KMT2C, MUC4, NEFH*, *PIK3CA*, and ZNF*814* (Fisher's exact test AdjP = value < 0.05) (Additional file 1: Table S7). In contrast, *TP53* mutations were prevalent in the ERPR + veHER2-ve subtype.

Several genes reported to be significant in breast cancer had enrichment of mutations in the triple-negative subtype. *TP53, CDC27, CTBP2*, and *PTEN* had recurrent mutations compared to their baseline rates (Additional file 1: Table S7). *TP53, PIK3CA, CRIPAK, CDC27, LZTR1, MUC16, and CBFB* are among the significantly mutated genes that could be classified as driver genes in the ER/PR-ve HER2 + ve subtype. A large number of genes (*n* = 155) were identified as driver genes in the ER/PR + veHER2-ve subtype compared to others. These significantly mutated genes contained breast cancer-associated genes, including *TP53, PIK3CA, CDC27, ESX1,* and *ESRRA*. Relatively fewer genes were identified as significantly mutated in ERPR + ve Her2 + ve and ER/PR-ve HER2 + ve subtypes (Additional file 1: Table S7). *TP53, LZTR,* and *CRIPAK* were identified as drivers in both ER/PR + ve Her2 + ve and ERPR-ve HER2 + ve subtypes. *TP53* was identified as a cancer driver in all subtypes.

Survival was negatively associated with mutations in *NBPF1* and positively associated with *MUC2* in triple-negative tumors **(**ZT4). In the ERPR + veHER2-ve subgroup, mutations in *TP53, GATA3, GPR98, MUC5B, NEB*, and *AHNAK2* were associated with shorter survival time, while mutations in *GATA3* were associated with longer survival. Additionally, mutations in *DSPP* and *GPR98* were found to increase the risk of metastasis in the ER/PR + ve HER2-ve subgroup.

Mutations co-occurred frequently in ER/PR + HER2-, while mutually exclusive mutations were rare (ZF5). *NBPF10* was mutually exclusive with *DNAH9*, *MUC5B*, *CDC27*, and *TTN*, and *TP53* mutations were mutually exclusive with *GATA3* and *CHD1*. Mutations in PIK3CA were mutually exclusive with *CRIPAK* mutations. In ER/PR + HER2+, co-mutations were linked to *MUC* genes, *TNXB*, and *DNAH17*, and mutations in *TP53* or *PIK3CA* were not found to co-occur (ZF5). ER/PR- HER2 + had few co-occurring mutations, and *TP53* mutations were mutually exclusive with *FOXQ1* and *CDC27*. No mutations were mutually exclusive in triple-negative subtype, but *MUC* genes, *AHNAK2*, *CROCC*, and *DNAH17* frequently co-occurred (ZF5).

ER/PR + veHER2 + ve and ER/PR-veHER2 + ve subtypes had higher percentages of samples with APOBEC signature (10.3% and 9%) compared to the rest of the subtypes. Each breast cancer subtype showed distinct mutational signatures. ER/PR-ve HER2 + ve subtype had SBS1 and SBS40, ER/PR + ve HER2-ve had four mutational signatures, including SBS13 (ZF6, 7), and triple-negative had four mutational signatures, with SBS40 unique to this subtype and SBS38 shared with ER/PR + ve HER2-ve (ZF8, 9).

### Comparison of mutation profiles across histological subtypes

Patients were divided into Invasive lobular carcinoma (ILC), Invasive ductal carcinoma (IDC), and Other histological types, with IDC being the most common (80%). Deleterious mutations were more prevalent in IDC, with higher frequencies observed in *PIK3CA*, *TTN*, *SVEP1*, *FAT1*, *ABCA13*, and *OBSCN* (Additional file 2: Fig. S5). Mutations in *MUC16*, *TP53*, *TTN*, and *AHNAK2* were more common in IDC, while *CDC27*, *PKHD1L1*, and *MUC21* had higher frequencies in ILC. ILC had more frequent mutations in *PIK3CA*, *CDH1*, *QRICH2*, *ABCA13*, *MUC12*, and *NEB* compared to the other subtypes.

**Fig. 4:**
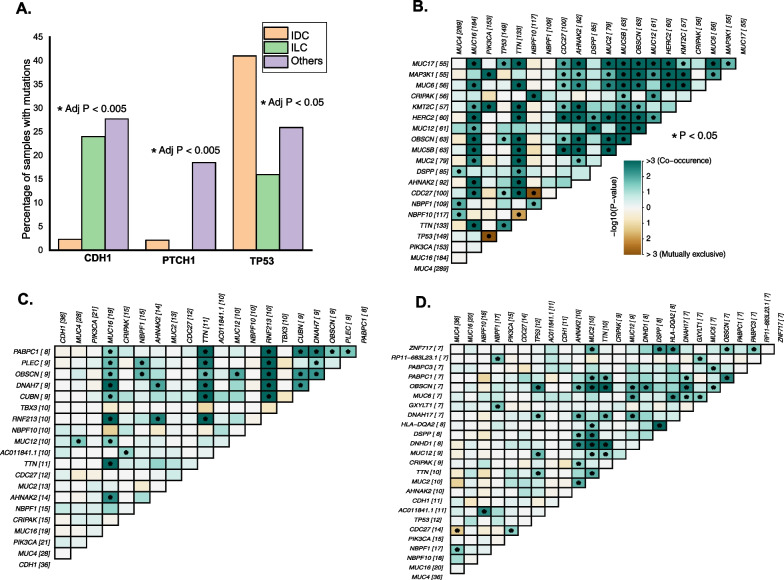
Distribution of mutations and representations of mutational interactions across histological subgroups. **A** Distribution of *CHD1*, *PTCH*1, and *TP53* mutations across the histological subtypes. **B**, **C**, **D** Mutational interactions identified across mutations in IDC, ILC, and Other. Green squares within the matrix indicate Co-occurrence, and the brown squares indicate mutual exclusivity across genes. The color scheme indicates the strength of the association, with darker colors indicating stronger co-occurrence or mutual exclusivity between the genes

TMB was higher in IDC than others, though the difference was not statistically significant (*T*-test *P* value ≤ 0.05) (Additional file 2: Fig. S6). Among the cancer-associated variants identified in the three subtypes, *CDH1*, *PTCH1,* and *TP53* mutation frequencies were significantly different (Fisher's test FDR corrected *P* value < 0.05) (Fig. 4A).

*TP53* mutations were frequent in the IDC subtype, whereas mutations in *CDH1* and *PTCH1* were relatively fewer in this subtype. *MutSigCV* identified a significant number of cancer driver genes in IDC compared to other subtypes. These included *PIK3CA, TP53, CDC27, GATA3, CBFB, ESX1, MAP3K1,* and *ESRRA* (Additional file 1: Tables S8–10). All of the oncogenic drivers identified in ILC were shared across all subtypes. *SF3B1* was identified as an oncogenic driver exclusive to the ‘other’ subtype. In all subtypes, four genes—*PIK3CA, RHPN2, CRIPAK*, and *CDH1*, were identified as cancer drivers (ZF10).

We identified 29 (6.8%) APOBEC-enriched samples among the IDC subtype. Several co-occurring mutations were identified in IDC, including *MUC17*, *MAP3K1, MUC6, HERC2, MUC5B, MUC16*, *CDC27*, and *KMT2C* (Fig. 3B). *PIK3CA* and *TP53* mutations along with *NBF10-CDC27* and *NBF10-TTN* mutations were mutually exclusive in IDC. We identified six samples in ILC (12%) that were APOBEC-enriched. Mutations in *MUC16, TTN*, and *RNF213* were found to be co-occurring with other mutations in ILC. Several co-occurring mutations were identified in the other subtype, including *TP53, MUC2, RNF213, DNAH7,* and *AHNAK2* (Fig. 3C, D). Mutations in *CDC27* and *MUC4* were mutually exclusive in the other subtype.

All mutational signatures identified in ILC and Other subtypes were shared between the two groups, whereas SBS2 and SBS29 were identified across all the three groups. Mutational signature linked to SBS38 and SBS13 was exclusively identified in IDC.

Mutations in *TP53, HERC2*, and *SYNE1* were associated with poor survival in the IDC subgroup (ZT5). We found mutations in *NCOR4* alone to confer a higher risk of poor survival in the ILC subgroup. Mutations in *NBPF* genes and *MUC4* were significantly associated with lower survival in the Other subtype. Several mutations in the IDC group were linked to relapse and metastasis events (ZT5). *NBPF* genes, *HERC2* and *MUC12*, were associated with relapse. Patients that developed distant metastasis had significantly higher mutations in *DNAH14, MUC4*, and *NBPF1*. *TP53* mutations were associated with both relapse and metastasis in IDC.

### Comparison of mutation profiles across tumor stages

The tumor samples were grouped into three stages based on the TNM staging system: stage 1, stage 2, and stage 3 and stage 4. The majority of patients in the cohort belonged to stages 1 and 2, with over 200 patients in each group, while 90 patients belonged to stage 3 and stage 4 at their initial breast cancer diagnosis. Several high-frequency mutations identified in stage 1 were deleterious, including MUC genes, *AHNAK2*, *MACF1*, *SVEP1*, *DNAH14,* and *SPEN* (Additional file 2: Fig. S7). Deleterious mutations in *OBSCN, MUC5B, MUC4, TP53, HRNR,* and *PARP4* were higher in stage 3 and stage 4.

*MutSigCV* identified a large number of cancer drivers in all stages. Several genes were identified as cancer drivers in tumor stages 1 and 2. *PIK3CA, TP53, CDC27, CDH1,* and *CRIPAK* genes were identified as drivers in all three stages (Additional file 1: Table S11). Stage 1 and stage 2 shared a number of driver genes, including *CBFB, GATA3, CTBP2, STK39, OVGP1, NBPF1,* and *PABPC1* (ZF11). Mutational interactions varied across all three stages. In stage 1 tumors, *NBPF10* mutations were mutually exclusive with *MUC17, MUC5B,* and *CDC27*. In stage 2 tumors, *PIK3CA* and *TP53* mutations were mutually exclusive, along with *NBPF10-TTN* mutations. Though *NBPF10* was mutually exclusive with several other mutations across different stages, in patients with stage 3 and 4, these mutations were mutually exclusive with mutations in *SPEN*. Several mutations coexisted with *TP53* mutations in stages 1 and 2, but not in stage 3 and 4. *PIK3CA* mutations coexisted with other mutations exclusively in stage 1.

Mutational signature analysis revealed both common and unique signatures across different stages. The signature associated with 'exposure to tobacco (chewing) mutagens' (SBS29), SBS5 (unknown etiology), and SBS38 (potential indirect damage from UV light) was identified in all three stages (ZF12). Signatures linked to SBS13 and SBS6 were identified exclusively in stage 2 (ZF12).

Patients with stage 1 tumors harboring *MAP3K1* mutations had a poor prognosis than wild type (*P* = 0.0013 HR = 2.67), whereas *PABPC1* and *NEFH* mutations provided a slight survival advantage (Additional file 2: Fig. S8). In stage 2 tumors, mutations in *PRUNE2* and *TP53* resulted in poor survival ((ZT6, Additional file 2: Fig. S9). *MUC2* mutations were associated with a favorable impact on survival (*P* value: 0.03; HR: 0.371) in this subgroup (Additional file 2: Fig. S9). In stage 3 and 4 tumors, *NBPF1* and *TP53* mutations resulted in poor survival (*P* value: 0.0045; HR: 2.56) (Additional file 2: Fig. S10). Mutations in *AHNAK2, NBOF10*, and *NBPF1* were associated with a higher risk of metastasis in stage 1. *PABPC3* and *LAMA5* mutations were associated with higher metastasis in stage 2. *DNAH14, CRIPAK, TP53, DNAH2, TTN, OBSCN, GPR98, NBPF1,* and *DSPP* mutants were associated with a higher risk of metastasis in stage 3. Similarly, *CRIPAK* and *MUC5B* were associated with a higher risk of relapse in stage 3.

### Comparison of mutation profiles across Ki67 levels

The study divided breast cancer tumors into Ki67-high and low groups based on expression levels and found that Ki67-high tumors had a higher percentage of mutations in *TP53, TTN, HRNR, MUC16*, and *AHNAK2* (Additional file 2: Fig. S11). The Ki67-low group had the highest percentage of mutations in *PIK3CA* and a lower percentage of *TP53* mutations. Average TMB and deleterious mutations were higher in the Ki67-high group than the Ki67-low group, except for *TP53* mutations (Additional file 2: Fig. S12). *TP53* (Fishers Adj *P* value < 0.000) was differentially mutated in Ki67 immunoreactivity groups, with higher mutations noted in patients with Ki67 high expression. *MutSigCV* analysis identified *CDC27, PIK3CA, GATA3, CDH1, CTBP2,* and *CRIPAK* as common cancer driver genes across both Ki67 expression groups (Additional file 1: Table S12, ZF13). In addition to these shared genes, several unique drivers were identified in each group-*CBFB, MAP3K1, RHPN2 MUC16,* and *TBX3* in Ki67-low, and *TP53, RBMX, PABPC1*, and *MTCH2* in Ki67-high.

Several mutations were identified in the Ki67-high expression group that co-occurred. These co-mutated genes included *TTN, MUC* genes, *CDC27, DNHD1,* and *CSPG4* (ZF14)*.* To note, the cancer-associated mutations in *TP53* co-occurred with *MUC4* mutations in this group. In contrast, *NBPF8* mutations were mutually exclusive with *MUC4* and *NBPF10* in the Ki67- high expression group. Relatively few mutations were co-occurring in the Ki67-low expression group, including *TTN, DNAH9, MUC16, MUC6, PIK3CA, KMT2C,* and *NBPF* (ZF14). Mutations in *PIK3CA* co-occurred with *KMT2C* mutations, whereas *NBPF10* and *TTN* mutations were mutually exclusive.

Common mutational signatures across all groups were identified as SBS29 (exposure to tobacco (chewing) mutagens) and SB5 (unknown etiology) (ZF15). However, Ki67-high expression tumors had exclusive mutational signatures of SBS13 (APOBEC Cytidine Deaminase (C > G) and SBS3 (defects in DNA-DSB repair by HR) (ZF15). On the other hand, Ki67- low expression group had exclusive mutational signatures associated with SBS6 (defective DNA mismatch repair) and SBS2 (APOBEC Cytidine Deaminase (C > T)) (ZF15).

Mutations in *NBPF1* were correlated negatively with survival in our cohort having Ki67-high expression. Metastasis risk increased with mutations in MUC6, whereas mutations in *AHNAK2* were protective (ZT7).

### Comparison of mutation profiles across tumors with different lymph node status

The study grouped tumors into two categories based on nodal involvement at diagnosis: NPlus (presence of nodal involvement) and N0 (absence of nodal involvement). About 58% of tumors were in the N0 category. Tumors in the N0 category had a marginally higher percentage of deleterious variants compared to NPlus tumors. *TP53* mutations were more frequent in the NPlus group, which had a higher percentage of samples with mutations in this cohort (Additional file 2: Fig. S13).

**Fig. 5:**
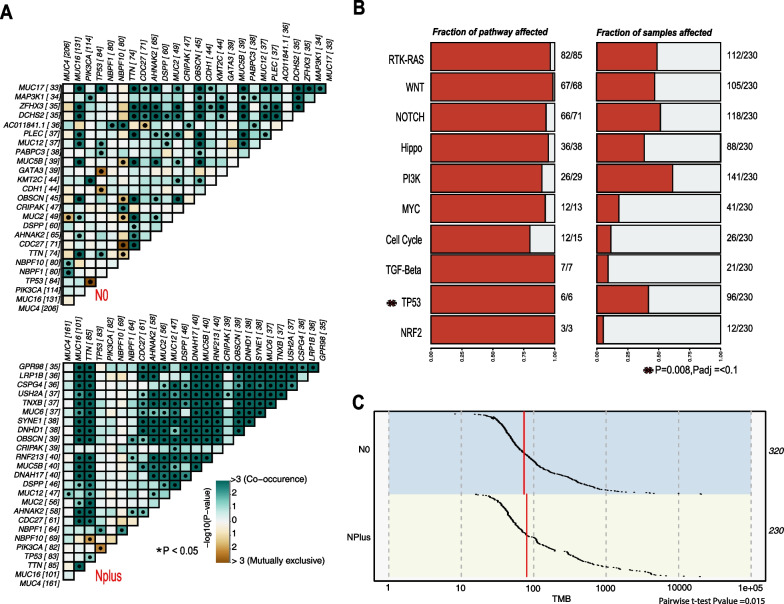
Distribution of mutational interactions across lymph node status. **A** Mutational interactions among Lymph node-positive (NPlus) and lymph node-negative (N0) tumors are represented here. Green squares within the matrix indicate Co-occurrence, and the brown squares indicate mutual exclusivity across genes. The color scheme indicates the strength of the association, with darker colors indicating stronger co-occurrence or mutual exclusivity between the genes. **B** Mutations identified candidate genes across oncogenic pathways in lymph node plus tumors are represented here. The percentage of mutated genes and samples mutated in each pathway are also represented. Asterisk indicates Fisher’s test *P* values for samples with *TP53* mutations in N0 and NPlus groups. **C** Tumor Mutation Burden across lymph node-negative and lymph node-positive tumors

*MutSigCV* analysis identified *TP53, PIK3CA, CDC27, PARP4,* and *MAP3K1,* as cancer drivers in both nodal involvement categories (Additional file 1: Table S13). Additionally, unique driver genes were identified in both N0 and NPlus groups. Mutations were found to be co-occurring more frequently in NPlus tumors than in tumors with no nodal status (Fig. 5A).

In the N0 category, mutually exclusive mutations were found in *TP53* and *NBPF10*, while in both categories, *TP53* and *PIK3CA* mutations were mutually exclusive. Mutations in genes belonging to 10 signaling pathways were compared, and the Nplus group had mutations in all genes in the *TGF-β, TP53*, and *NRF2* pathways (Fig. 5B). A higher percentage of mutations in *TP53* pathway genes was found in the Nplus group compared to N0 (*t*-test *P* value = 0.008). TMB was marginally higher in tumors with nodal involvement (*t*-test *P* value 0.01, Fig. 5C).

Analysis of mutational signatures revealed shared signatures for SBS29 and SBS6 in both N0 and NPlus categories (ZF16), along with several unique signatures.

Patients with *KMT2C* mutations in lymph node-negative tumors and *TP53* mutations in lymph node-positive tumors had lower survival than those with wild-type tumors (Additional file 2: Fig. S14, ZT8). *DNHD1* mutations were associated with a slight survival advantage for lymph node-positive tumors. Mutations in *CRIPAK* and *MUC12* were associated with a lower risk of relapse in lymph node-negative tumors (ZT3), and CRIPAK mutations were also protective against metastasis in both N0 and NPlus groups. However, *TP53* mutations were associated with the risk of metastasis in lymph node-positive tumors (ZT3).

### Comparison of mutation profiles in Caucasian versus African Americans

More than 94% of our patient cohort was Caucasian.

**Fig. 6:**
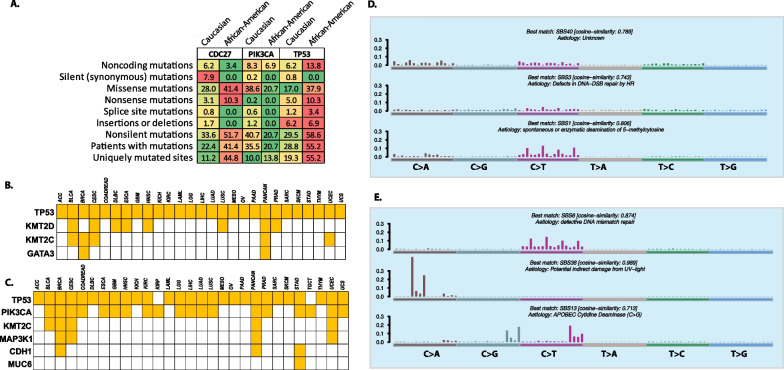
**A** Distribution of mutations in *CDC27, PIK3CA,* and *TP53* genes in Caucasians and African American patients are represented here. Green squares within the matrix indicate Co-occurrence, and the brown squares indicate mutual exclusivity across genes. The color scheme indicates the strength of the association, with darker colors indicating stronger co-occurrence or mutual exclusivity between the genes. **B**, **C** Reported TCGA drivers identified in Caucasians and African American patients are represented here. Yellow squares indicate if the gene was identified as an oncogenic driver. **C**, **D** Unique mutational signatures identified in Caucasians and African American patients

*MutSigCV* analysis identified shared and unique cancer driver genes among different races (Additional file 1: Table S14). The analysis identified three genes (*TP53, CDC27*, and *CDH1*) as cancer drivers in African Americans, while 194 genes were identified in Caucasians. *TP53* and *CDC27* had a higher mutation frequency in African Americans compared to Caucasians (Fig. 6A).

Several drivers identified among TCGA cancer cohorts were also identified in both ethnicities. *TP53* and *KMT2C* genes were identified as drivers in both groups. *KMT2D* and *GATA3* were uniquely identified in African Americans as cancer drivers reported in TCGA data (Fig. 6B), whereas *PIK3CA, MAP3K1, CDH1,* and *MUC6* were identified in Caucasians (Fig. 6C).

APOBEC-enriched samples were highest among Caucasians (8.8%), and no APOBEC enrichment was noted among African Americans. Mutational signatures associated with SBS5 and SBS29 were identified in both groups along with several unique signatures (Figs. 5E, 6D).

Caucasians had an abundance of co-occurring mutations compared to African Americans (ZF17). Mutations involving *MUC16, MUC6, MUC2, MUC12, MAP3K1, KMT2C, CDC27,* and *AHNAK2* co-occurred in Caucasians (ZF10). Mutually exclusive mutations among *TP53, CDH1, PIK3CA,* and *NBPF10* were frequent in Caucasians. In African Americans, very few co-occurring mutations were identified, along with one mutually exclusive mutation between *TP53* and *GATA3* (ZF17).

Survival analysis identified mutations in *NBPF1, AHNAK2, NBPF10* associated with lower survival in African Americans. On the other hand*, MUC5B,* and *MAP3K1* showed a significant negative association with overall survival (ZT9). *TP53* mutations confer a higher risk of relapse and metastasis in Caucasians (ZT9).

## Discussion

This study analyzed the somatic mutational profile of 554 breast cancer patients and is characterized by integrating genomic variants along with long clinical follow-up. This cohort represents the population structure of the US Midwest, with a higher representation of Caucasians than any other racial or ethnic community. About 31% of the patients had at least one first- or second-degree relative diagnosed with breast or ovarian cancer, suggesting a strong familial risk factor in the etiology of this cancer.

*MUC4*(68%)*, MUC16*(45%)*, PIK3CA*(36%)*, TTN*(31%)*, TP53*(30%)*, NBPF10*(30%)*, NBPF1*(29%)*, CDC27*(26%)*, AHNAK2*(24%), and *MUC2*(22%) were the top mutated genes in our breast cancer cohort. Of these genes, mutations in *PIK3CA, TTN, TP53, and MUC16* were also highly represented in the TCGA breast cancer database (TCGA PanCancer Atlas data, cBioPortal). TMB varied across several subgroups in our cohort. For example, the triple-negative subtype had significantly higher TMB than other subtypes. On the other hand, TMB status was higher in grade 3 tumors, Ki67-high tumors, IDC subtype, and among African Americans, though not statistically different. However, several subgroups mentioned above have a higher percentage of deleterious mutations in cancer-associated genes, which could be functionally relevant in late-stage or aggressive cancers. Higher TMB has been associated with a better prognosis with immune checkpoint inhibitors and is now investigated as a predictive biomarker [23, 24]. TMB high breast tumors have also been associated with unique mutational profiles and enriched with actionable mutations, which provides new opportunities for innovative therapeutic approaches [25].

Mutual exclusion of mutations in *PIK3CA* and *TP53* was very pronounced when patients were classified across grades. *PIK3CA* mutations were prevalent in grade 1 tumors, whereas *TP53* mutations were higher in grade 3 tumors. Both *PIK3CA* and *TP53* mutations showed opposite trends across grades (Fig. 2). Biological processes that are similar in nature can exhibit mutual redundancy, and a single alteration is adequate to disrupt the function of the process. Identifying instances of mutations that are mutually exclusive can help identify unknown functional, synthetic lethal interactions. In contrast, mutations that co-occur can be synergetic leading to malignancy and treatment response [26]. Significant associations of *PIK3CA* mutations to lower grade and smaller size of breast tumors were also reported in a meta-analysis containing 19 individual studies [27]. About 36% of the patients had *PIK3CA* mutations; the distribution of *PIK3CA* mutations across all subsets is represented in Fig. 3A.

*PIK3CA* mutation frequency was also significantly different across subtypes based on ER/PR status. ERPR + veHER2-ve patients had the highest percentage of *PIK3CA* mutations, and ERPR-veHER2 + ve had the lowest. Earlier studies have identified the prognostic potential of *PIK3CA* mutations, often associated with better clinical response [27–29]. In addition, *PIK3CA* mutations have also been linked to cancer initiation through ER signaling [30, 31].

*TP53* mutations are harbored by most cancers and are also associated with therapeutic resistance and poor prognosis in various cancers [32, 33]. The overall somatic mutation rate of *TP53* in our cohort was 30%. Mutations identified in our cohort are represented in Fig. 3B. *TP53* mutation frequency was lowest in grade 1 and highest in grade 3 tumors (FDR corrected *P* value < 0.05, Fig. 2). Similar to *PIK3CA, TP53* mutations were also significantly different across subtypes based on ER/PR status, with the highest mutation rate in triple-negative subtype and lowest among ERPR + veHER2-ve tumors. A recent report from a Chinese breast cancer cohort found that the association of *TP53* mutations to pathological grade is consistent with our observations [34].

**Fig. 7:**
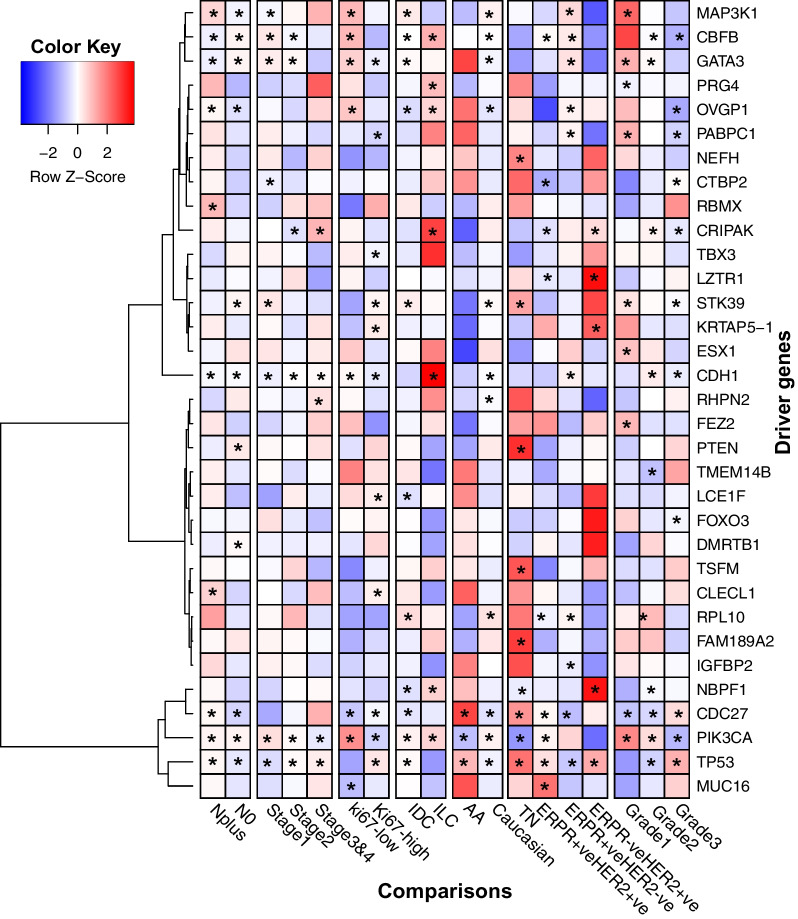
Distribution of driver genes and mutation frequencies identified across different comparisons. Stars represent driver genes that were identified as significantly mutated. The red color indicates higher patient frequencies with mutated driver genes, and the blue indicates lower mutational frequencies of driver genes

Several driver genes were identified in this cohort. Several genes, including *TP53, PIK3CA, CDC27, CDH1, STK39, CBFB, MAP3K1,* and *GATA3,* were identified across different comparison groups as the top-ranked driver genes, in addition to few unique drivers (Fig. 7).

These genes include *IGFBP2* in ERPR + veHER2-ve tumors, *TSFM* in triple-negative tumors, *FEZ2* in ERPR-veHER2 + ve tumors, and *TBX3* in tumors with Ki67 expression. *GATA3* is a transcriptional factor critical for breast development and is associated with luminal transcription in breast cancer [35]. Loss of *GATA3* expression leads to the dedifferentiation of luminal epithelial cells, leading to cancer progression and metastasis [36]. The somatic mutation rate of *GATA3* was 13% in our cohort (Fig. 3C). *GATA3* expression is correlated to estrogen receptor alpha expression and better prognosis and is frequently mutated in breast cancer [37–39]. Mutations in *GATA3*, particularly in the DNA binding domain, caused altered transcription factor localization leading to dedifferentiation [40].

Serine/Threonine Kinase 39 (*STK39*) regulates osmotic stress responses, and reports have linked lower expression to treatment resistance in breast and prostate cancers [41, 42]. *STK39* has also been implicated in several other cancers, including lung, osteosarcoma, and renal carcinoma [43–45]. In breast cancer, *STK39* is reported as an early antigen, and its expression was associated with poor prognosis [46]. A recent report has identified the involvement of *STK39* in breast cancer progression and metastasis [47]. Our study identified *STK39* as a driver in patients with stage I tumors and those with no nodal involvement (N0). Tumors with high Ki67 expression, IDC subtype, or tumors of TN subtype also had higher mutations in *STK39*.

*CBFB* (Core-Binding Factor Subunit Beta), a transcriptional co-factor for RUNX proteins, is mutated in 6.7% of our BC cohort (4% reported in TCGA PanCancer Atlas dataset, CBioPortal). *CBFB/RUNX1* axis is reported to function as a tumor suppressor in breast cancer [48, 49]. *TP53* and *CBFB* mutations were mutually exclusive, and their association has been shown to interact with *TAp73* expression, which acts as a tumor suppressor in breast cancer [50]. In addition, *CBFB* is also shown to bind to a large pool of mRNAs and enhance translation in breast cancer cells [48]. We identified *CBFB* as a cancer driver in most subgroups in our breast cancer patients.

Investigation of the prognostic value of gene mutations revealed that *TP53* mutations were significantly correlated with lower survival in stage 2, stage 3 and 4, Nplus, ERPR + veHER2-ve, and IDC subgroups (Additional file 2: Fig. S15). *NBPF1* mutations were associated with shorter survival in stage 3 and 4, Ki67-high, TN, and African Americans in our cohort. *NBPF1* (Neuroblastoma Breakpoint Family, member 1) is a tumor suppressor gene associated with several cancers, including gastric cancer and neuroblastoma [51, 52]. Functional studies in cervical cancer cell lines identified *NBPF1* regulation of cell invasion and apoptosis by activating PI3K/mTOR signaling pathways, which are key mechanisms in cancer progression [53]. The role of *NBPF1* in breast cancer is still unknown, though it is reported that *NBPF1* mutations in noncoding regulatory regions are higher in breast cancer patients [54], along with hypermethylation [55]. Additional studies to characterize *NBPF1* function and recurrent mutations in breast cancer are warranted.

*MUC5B* mutations were associated with a higher risk of death in grade 2 and ERPR + veHER2-ve subgroups. *MUC2* mutations, on the other hand, provided a survival advantage among stage 2 and triple-negative BCs in our cohort. Mucins are O-glycosylated proteins expressed at the surface of epithelial cells and are involved in epithelial differentiation, cell signaling, cell adhesion, invasion, and growth [56]. Mucins are associated with tumor cell differentiation and proliferation through ligand–receptor interactions and morphogenetic signal transduction [56]. *MUC5B* expression is disrupted in breast cancers and is associated with increased cell proliferation and metastasis of breast cancers and can be explored as a cancer biomarker and a therapeutic target [57, 58]. *MUC5B*, along with other mucins, is also associated with 5-FU and cisplatin resistance [59]. *MUC* gene expression and recurrent mutations in BC subgroups should be investigated further, given the prognostic value of MUC genes.

*PAPBCI, NEFH, DNHD1, GATA3, GPR98,* and *ACO11841.1* confer a prognostic advantage in several BC subgroups. *GPR98* was associated with a higher risk of metastasis in stage 3 and 4, ERPR + veHER2-ve subgroups, and in Caucasian patients (Additional file 2: Fig. S16). *GPR98* belongs to a family of adhesion GPCRs that are less explored in breast cancer. These adhesion GPCRs are involved in several functions like cell adhesion, cell motility, cell guidance, and tumor cell interactions [60–62]. *GPR98, a.k.a ADGRV1,* has a reported mutation frequency of 2% in the TCGA breast cancer cohort and is one among the frequently mutated GPCR in TCGA cancers [63]. There are no additional reports of *GPR98* mutations in breast cancer, and the functional consequence of these mutations in breast cancer is unknown.

Several mutational signatures were identified across different BC subgroups in our study. These mutational signatures are left behind specific patterns ‘signatures’ correlated with DNA damage repair defects, exposure to carcinogens, or combinations of structural variants that can be extracted using matrix decomposition algorithms such as NMF. Mutational signatures including SBS29 (Aetiology: exposure to tobacco (chewing) mutagens), SBS5 (Aetiology: Unknown), and SBS38 (Aetiology: Potential indirect damage from UV-light) were identified across most of our comparison groups (ZT10). For instance, several interesting mutational signatures, SBS3 representing ‘Defects in DNA-DSB repair by HR’ was identified in cancers with Ki67-high expression and lymph node-positive cancers. These mutational signatures are reported to be associated with characteristic changes in tumor histology, gene expression, or gene mutations [64].

This study presents results from a large breast cancer cohort from the US Midwest. Though this study is sufficiently large, the percentage of several subgroups within this cohort is underrepresented. For example, the African American population in this cohort is extremely low, reflecting the local population structure. In addition, only 17% of the breast cancer patients in this cohort have developed recurrence. These factors should be considered when analyzing the genomic characteristics of these underrepresented patient-specific groups.

## Conclusion

We report here a large, well-characterized breast cancer cohort from the US Midwest. Somatic variants identified across different patient groups were analyzed, and several variants related to different subgroups were identified. For example, in our cohort, mutations in *PIK3CA* and *TP53* were mutually exclusive, and this was pronounced across the different grades. Grade 1 tumors had higher *PIK3CA* mutations, whereas grade 3 tumors had higher *TP53* mutations. In addition to the characteristic mutations associated with each subtype, we also identified several driver mutations, including *TP53, PIK3CA, CDC27, CDH1, STK39, CBFB, MAP3K1*, and *GATA3,* across different groups. Further studies on understanding the functional relevance of these mutations in BC oncogenesis are warranted.

## Materials and methods

### Patient samples and clinical information

Tumor and germline samples were collected from 554 patients. Tumor tissue collected was either FFPE preserved, frozen, or fresh. This study was approved by the Institutional Review Board (IRB) of the University of Nebraska Medical Center (0155-13-EP, and the IRB for the data collection (iCaRe2) is 253-13-EP). Patients gave their informed written consent to participate in this study. All paired tumor and germline specimens were processed using whole-exome sequencing to identify somatic variants that include single nucleotide variants (SNVs) and insertions/deletions (indels). Patient characteristics are depicted in Table 1. Patients were further classified according to different biological and tumor-specific parameters, including grade, stage, hormone receptor status, histological subtype, Ki67 expression, lymph node status, and race. Three patients presented with bilateral breast cancers, and each cancer was sequenced separately, though only one representative tumor was included in this study. All patients enrolled in this study were followed up for a median follow-up time of 7.6 years.

### Tissue collection and sample processing

Oncologists and pathologists from all participating sites worked collaboratively to select breast cancer patients for the whole-exome DNA sequence study. The initial Fred and Pamela Buffett Cancer Center (FPBCC) or external site review of breast cancer specimens determined (1) whether there was an adequate number and percentage (> 35%) of tumor cells in the specimen; (2) if the specimen would be appropriate for the whole-exome DNA sequence analysis, and (3) if a corresponding patient white blood cell (WBC) specimen collected through the FPBCC’s iCaRe2 Breast Cancer Registry or confirmed adjacent normal (unaffected) tissue was available for germline analysis. FFPE tissue blocks passing initial external site review were sent to FPBCC pathology for centralized local review. Approved specimens were processed by the UNMC Tissue Science Facility for the preparation of 10-micron unstained tissue sections embedded on glass slides. DNA isolation from corresponding patient germline WBC and FFPE tumor (or normal adjacent germline) specimens was performed by the FPBCC Molecular Biology/High-Throughput Screening Facility. DNA was extracted and purified from WBC or fresh frozen and FFPE samples using QIAamp DNA Mini and QIAamp DNA FFPE Tissue (QIAGEN) kits, respectively, as per the manufacturer’s instructions. Purified DNA isolates were quantified by Nanodrop 2000, followed by double-stranded DNA assessment using Qubit (3.0) dsDNA HS Assay kit (Invitrogen), adjusted to a standardized volume of 50 µl and stored at -80̊C.

### Whole-exome sequencing

Whole-exome sequencing (WES) was performed on DNA extracted from paired tumor-germline samples from all patients. The samples were processed as follows: 200 ng of genomic DNA of each sample was used as the starting material and processed using the Agilent SureSelect XT/ Clinical Research Exome kit as per the recommended procedure. Prepared libraries were then sequenced with a paired end read length of 150 bp on an Illumina HiSeq2500 sequencer using HiSeq V3 reagents. WES data in FASTQ format were processed to remove adapters, unknown terminal bases (Ns), and low-quality 3’ regions (Phred score < 30) using *fqtrim* (http://ccb.jhu.edu/software/fqtrim/, DOI-https://doi.org/10.5281/zenodo.593893). The quality of trimmed reads was assessed using *FastQC* [65], and those passing *FastQC* assessment were aligned to the human reference genome (hg19) with *Borrows-Wheeler Aligner* (v.0.7) [66]. The aligned reads were further processed through the *GATK* pipeline [67, 68] for base quality score recalibration, INDEL (insertions and deletions) realignment, and mark duplicates, according to GATK’s best practices recommendations [68, 69]. Four variant callers, *MuTect* (v.3.1) [70], f*reebayes* (v1.1.0-4) [71], *VarDict* [72], and *VarScan* (v.2.4) [73] were used to take both tumor and normal/germline bam files as input and to call somatic variants. The ensemble method was employed to identify somatic variants that were called by at least two of the four variant callers. These analyses were performed using the cancer variant calling pipeline incorporated in the *bcbio-nextgen* python toolkit (https://github.com/chapmanb/bcbio-nextgen). The VCF (variant call format) files generated from the pipeline were further annotated using ANNOVAR [74] and VEP [75] to identify the deleterious consequences of the genomic variations on splice junctions, protein structure and function, downstream effects on signaling pathways, and gene/protein interaction networks.

### Mutation analyses

Synonymous mutations and variants falling into intronic and untranslated regions were not considered in this analysis. Frequently mutated genes in each study group (based on seven different clinical, histological, and demographic parameters) were analyzed and compared. *MutSigCV* algorithm at default settings was used to identify significantly mutated genes compared with background mutation rates in each subgroup [76]. In addition to identifying driver genes using *MutSigCV*, we also identified cancer-associated variants using *Cancer Hotspots* [77], *CScape* [78], *CHASMplus* [79], *Mutpanning* [80], *Clingen* [81], *Clinvar* [82], *GRASP* [83], and *GWAS Catalogue* [84]. For *CHASMplus*, we selected only those variants that are linked to breast cancer (CHASMplus BRCA) and selected variants with a P-value cut off of 0.05. For *CScape*, variants with a cutoff score of 0.89 were chosen as oncogenic. *Mutpanning* identifies cancer driver genes by modeling the mutation probability of each genomic position depending on its neighboring nucleotide architecture and background mutation rate. A Q-value cutoff of 0.05 was used to determine if a gene variant was oncogenic. For *Clingen, Clinvar, GRASP*, and *GWAS Catalogue*, a gene variant was selected as oncogenic if their curated database provided evidence for cancer association.

We also classified variants as deleterious or pathogenic using several algorithms, including *Funseq2* [85], *DANN* [86], *ALoFT* [87], *CADD* [88], *FATHMM* [89], *MetaLR* [15], *MetaSVM* [15], *PhD-SNPg* [90], *REVEL* [91], and *VEST* [92]. The python package, *openCRAVAT,* was used to perform all the genomic variant interpretations for cancer-associated and deleterious variants [93]. Tumor Mutation Burden (TMB) was calculated for each sample using the total number of non-synonymous variants with functional impact divided by the length of the mega-base in coding regions captured with the exome sequencing. Survival analysis was performed for mutations identified in our analysis across all groups using the Kaplan–Meier survival analyses implemented in the R/Bioconductor package, *Maftools* [94]. A two-sided *P* < 0.05 was considered statistically significant in this analysis. Mutational patterns including mutual exclusivity and co-occurrence were also determined for top 25 mutated genes in each group using *Maftools.*

Mutations in important oncogenic signaling pathways identified to be frequently mutated in cancer were also profiled across different groups in our study [95]. This study analyzed key candidate genes in each of these oncogenic pathways curated based on TCGA mutation profiles, literature review, and databases [95].

### Mutation signature analyses

Mutation signature analyses were performed using *Maftools *[94] that extracts the 5′ and 3′ bases adjacent to the mutation and creates a 96 × sample size count matrix using the ‘trinucleotideMatrix’ function. The ‘*extractSignatures’* function in *Maftools* uses NMF (non-negative matrix factorization) to factorize this count matrix to identify the optimal rank r. Mutational signatures identified through matrix factorization were compared to well-characterized and annotated signatures in the COSMIC database [provide reference].

### APOBEC enrichment analyses

APOBEC signature is one of the most prominent mutation signatures in cancer, present in half of the human tumors. Signatures of APOBEC cytidine deaminase DNA-editing-enriched samples in our cohort were identified using *Maftools*, which calculates an enrichment score associated with the APOBEC-related mutagenic processes in each sample by comparing the *C* > *T* mutations within the tCw motif among ± 20 nucleotides surrounding each mutated cytosine to the background [96]. Samples were classified into APOBEC-enriched (enrichment score > 2) and non-APOBEC-enriched (enrichment score < 2). Genes overrepresented in the APOBEC-enriched samples were identified using one-way Fisher's exact test.

## Supplementary Information

**Additional file 1. Supplementary Tables S1-S14. Table S1:** Cancer associated variants differentially mutated across tumor grade. **Table S2:** Co-occuring or mutually exclusive mutations in Grade1. **Table S3:** MutSig CV results Grade1. **Table S4:** Co-occuring or mutually exclusive mutations in Grade2 tumors. **Table S5:** MUtSig CV results Grade2. **Table S6:** MUtSig CV results Grade3. **Table S7:** Driver genes identified by MutSigCV across tumor subtypes. **Table S8:** MUtSig CV results IDC. **Table S9:** MUtSig CV results IMC. **Table S10:** MutSig CV results others. **Table S11:** MutSigCV results across Tumor stage. **Table S12:** MutSigCV results Ki67. **Table S13:** MutsigCV driver genes associated with Node status. **Table S14:** MutSigCV results-Ethnicity.

**Additional file 2**. **Figure S1**: Tumor Mutation Burden compared between grade 1, 2 and 3 breast cancer patients. The redline indicates median TMB. **Figure S2**: Kaplan–Meier survival analyses stratified by CBFB, HERC2 and TP53 gene mutations. **Figure S3**: Tumor Mutation Burden compared between ER/PR+veHER2-ve, ER/PR+veHER2+ve, ER/PR-ve HER2+ve, and Triple-negative breast cancer patients. The redline indicates median TMB. **Figure S4**: Distribution of patient cohort across the hormonal subtypes. The upper panel of the figure provides the distribution of tumor mutation burden for each patient assigned to ER/PR+veHER2-ve, ER/PR+veHER2+ve, ER/PR-ve HER2+ve, or Triple-negative. Patient characteristics, including vital status, family history, subtype status, and age, are also included for each group. The bottom panel represents the top mutated genes across the three groups. The percentage of deleterious variants in each gene is also represented, along with the type of mutation detected. **Figure S5**: Distribution of patient cohort across the histological subtypes. The upper panel of the figure provides the distribution of tumor mutation burden for each patient assigned to Invasive lobular carcinoma (ILC), Invasive ductal carcinoma (IDC), and Other. Patient characteristics, including vital status, family history, subtype status, and age, are also included for each group. The bottom panel represents the top mutated genes across the three groups. The percentage of deleterious variants in each gene is also represented, along with the type of mutation detected. **Figure S6**: Tumor Mutation Burden compared across the histological subtypes. The redline indicates median TMB.

## Data Availability

All data generated or analyzed during this study are included in this published article (and its Supplementary Information files). Whole-exome sequencing Fastq files are uploaded in the NCBI SRA database with BioProject accession number: PRJNA824495. Additional figures (ZF) and tables (ZT) that were not included with the manuscript are available in the ZENODO repository https://doi.org/10.5281/zenodo.8122769
