# Supplementary material for: The mutational landscape of a US Midwestern breast cancer cohort reveals subtype-specific cancer drivers and prognostic markers
Source: Hum Genomics. 2023 Jul 15;17:64. doi: 10.1186/s40246-023-00511-6 (PMC10349437; doi:10.1186/s40246-023-00511-6)
Supplement: Supplementary file 2 — Additional file 2. Figure S1: Tumor Mutation Burden compared between grade 1, 2 and 3 breast cancer patients. The redline indicates median TMB. Figure S2: Kaplan–Meier survival analyses stratified by CBFB, HERC2 and TP53 gene mutations. Figure S3: Tumor Mutation Burden compared between ER/PR+veHER2-ve, ER/PR+veHER2+ve, ER/PR-ve HER2+ve, and Triple-negative breast cancer patients. The redline indicates median TMB. Figure S4: Distribution of patient cohort across the hormonal subtypes. The upper panel of the figure provides the distribution of tumor mutation burden for each patient assigned to ER/PR+veHER2-ve, ER/PR+veHER2+ve, ER/PR-ve HER2+ve, or Triple-negative. Patient characteristics, including vital status, family history, subtype status, and age, are also included for each group. The bottom panel represents the top mutated genes across the three groups. The percentage of deleterious variants in each gene is also represented, along with the type of mutation detected. Figure S5: Distribution of patient cohort across the histological subtypes. The upper panel of the figure provides the distribution of tumor mutation burden for each patient assigned to Invasive lobular carcinoma (ILC), Invasive ductal carcinoma (IDC), and Other. Patient characteristics, including vital status, family history, subtype status, and age, are also included for each group. The bottom panel represents the top mutated genes across the three groups. The percentage of deleterious variants in each gene is also represented, along with the type of mutation detected. Figure S6: Tumor Mutation Burden compared across the histological subtypes. The redline indicates median TMB. [file 40246_2023_511_MOESM2_ESM.pdf]

Supplementary Figure 1

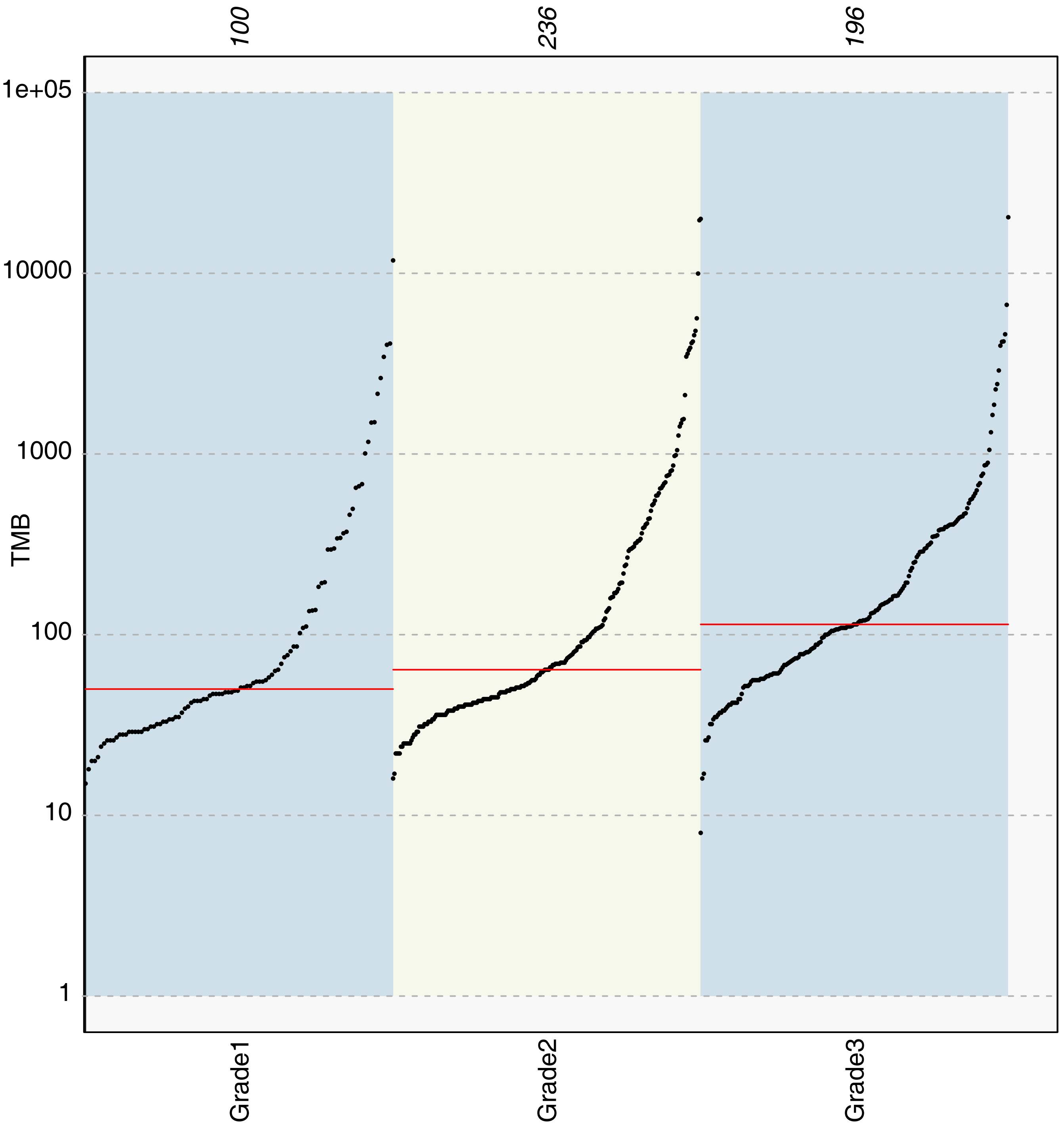

Supplementary Figure 2

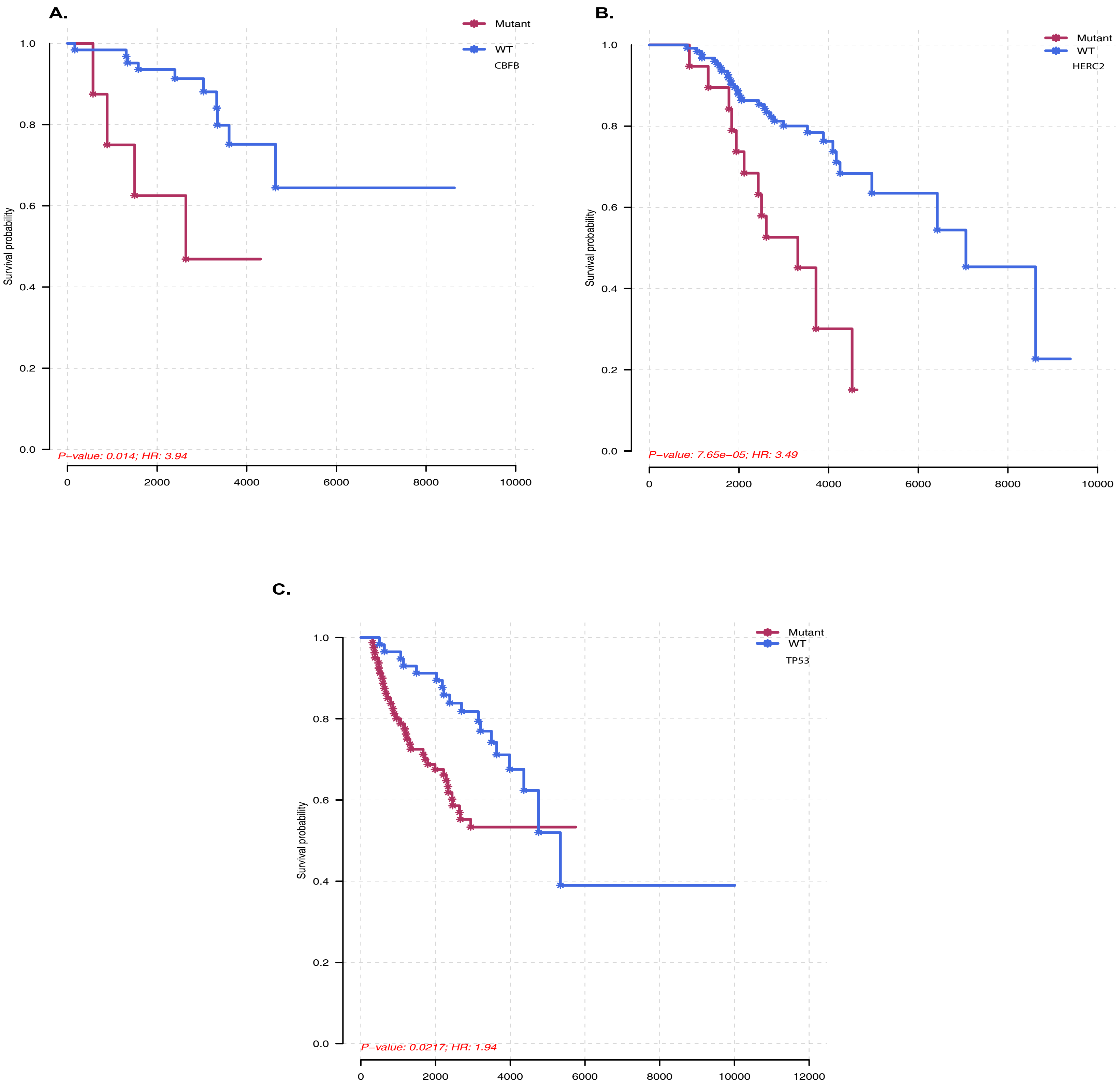

Supplementary Figure 3

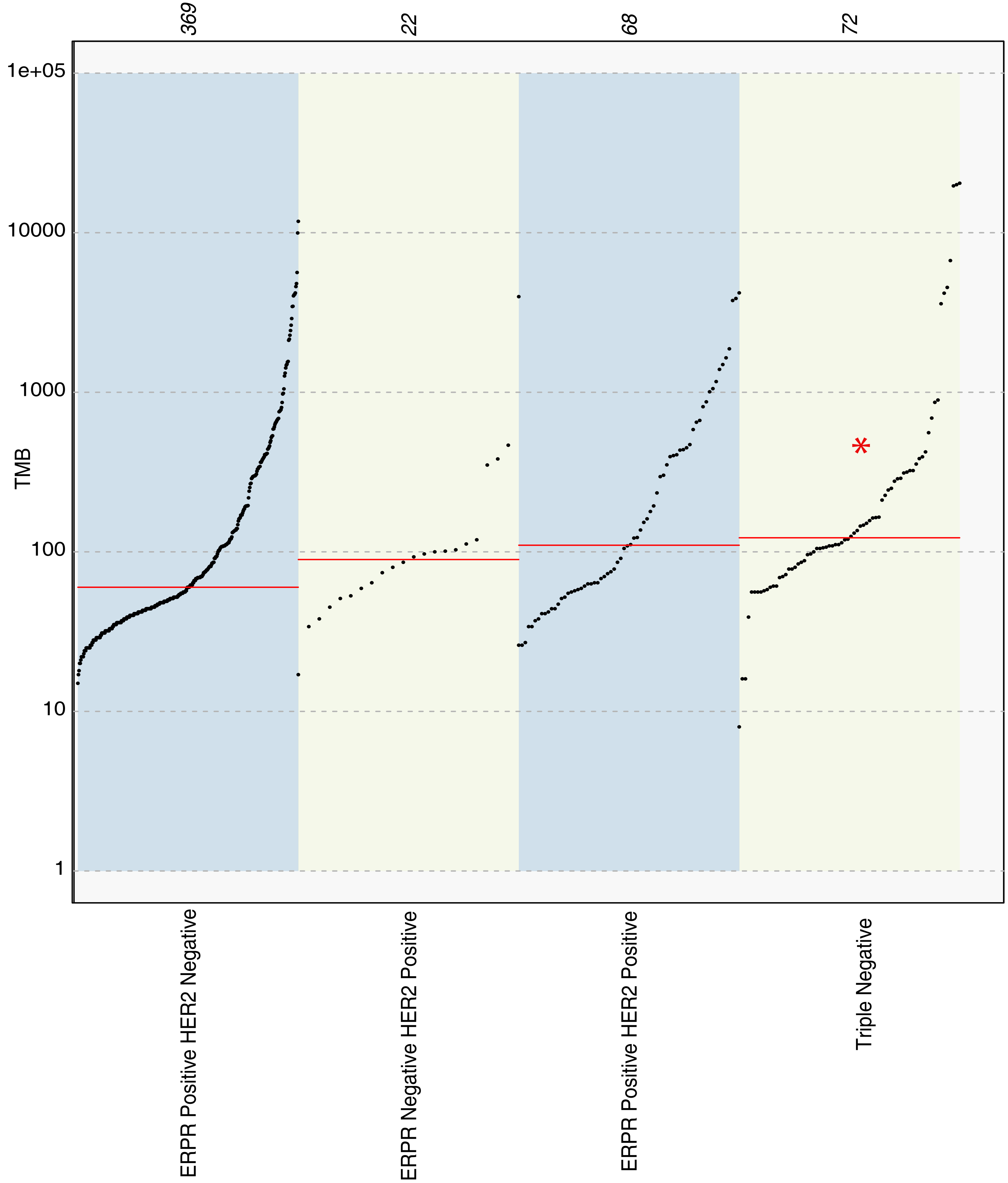


* T-test <0.005 across all comparisons

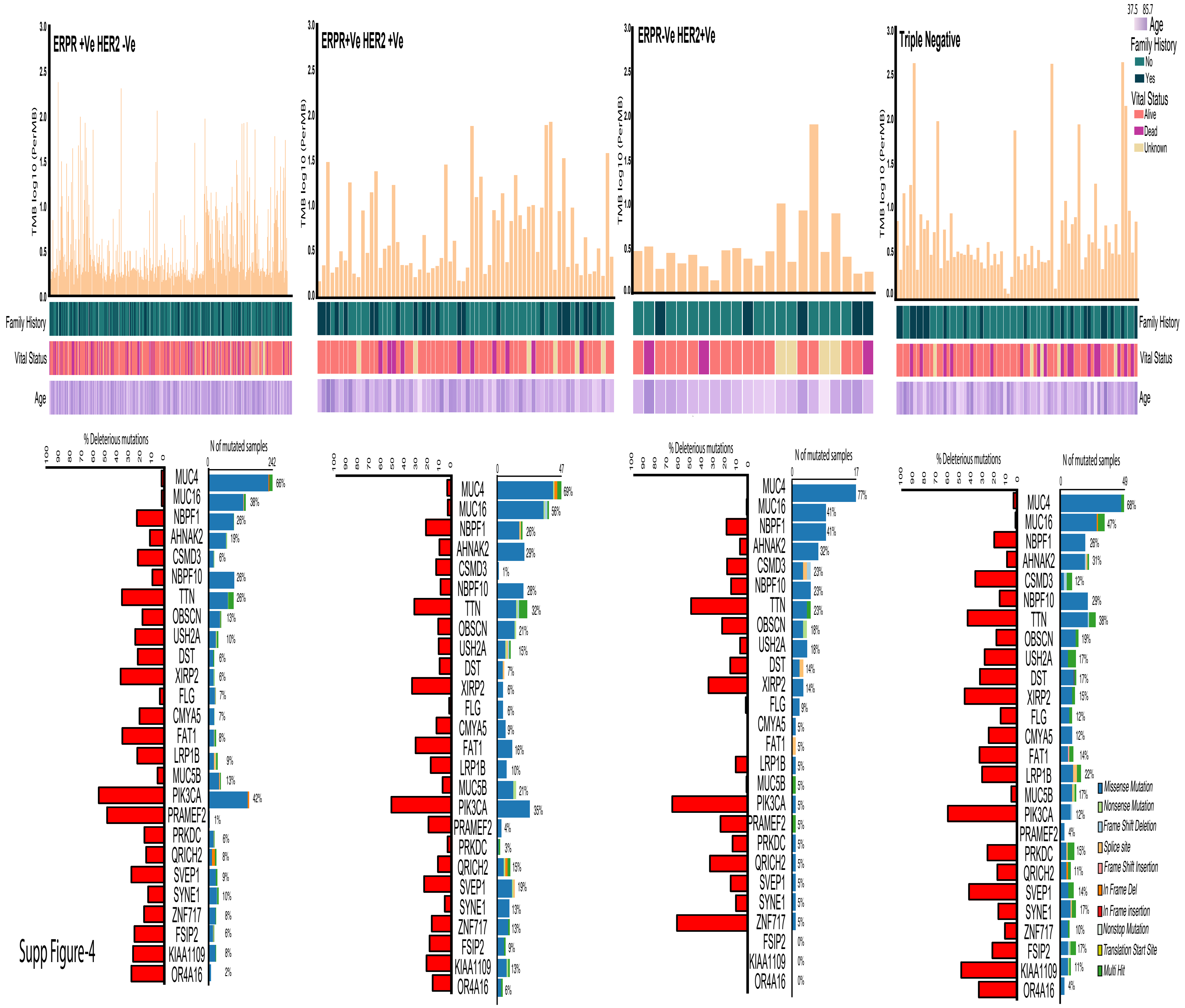

Supp Figure-4

Supp Figure 5

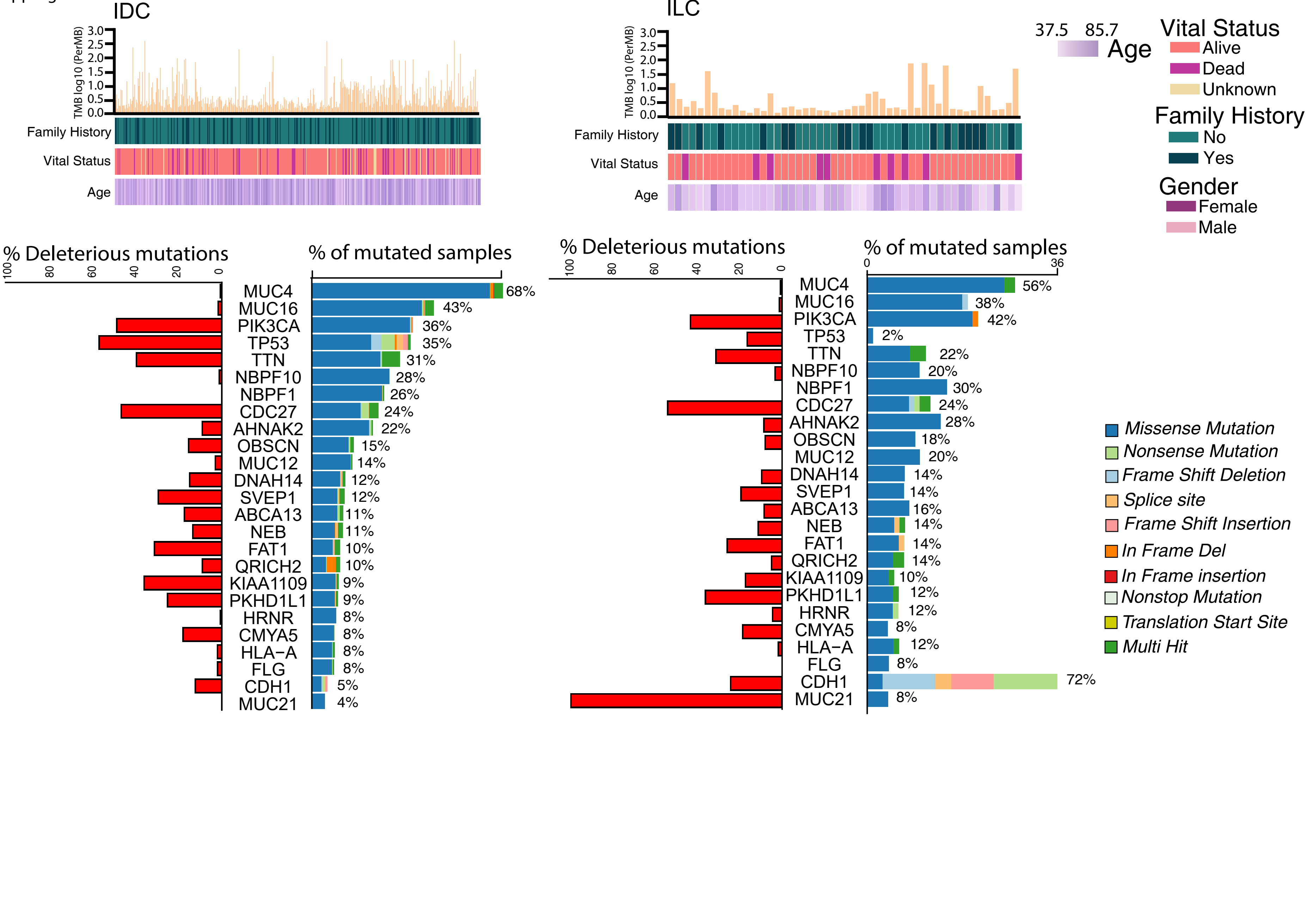

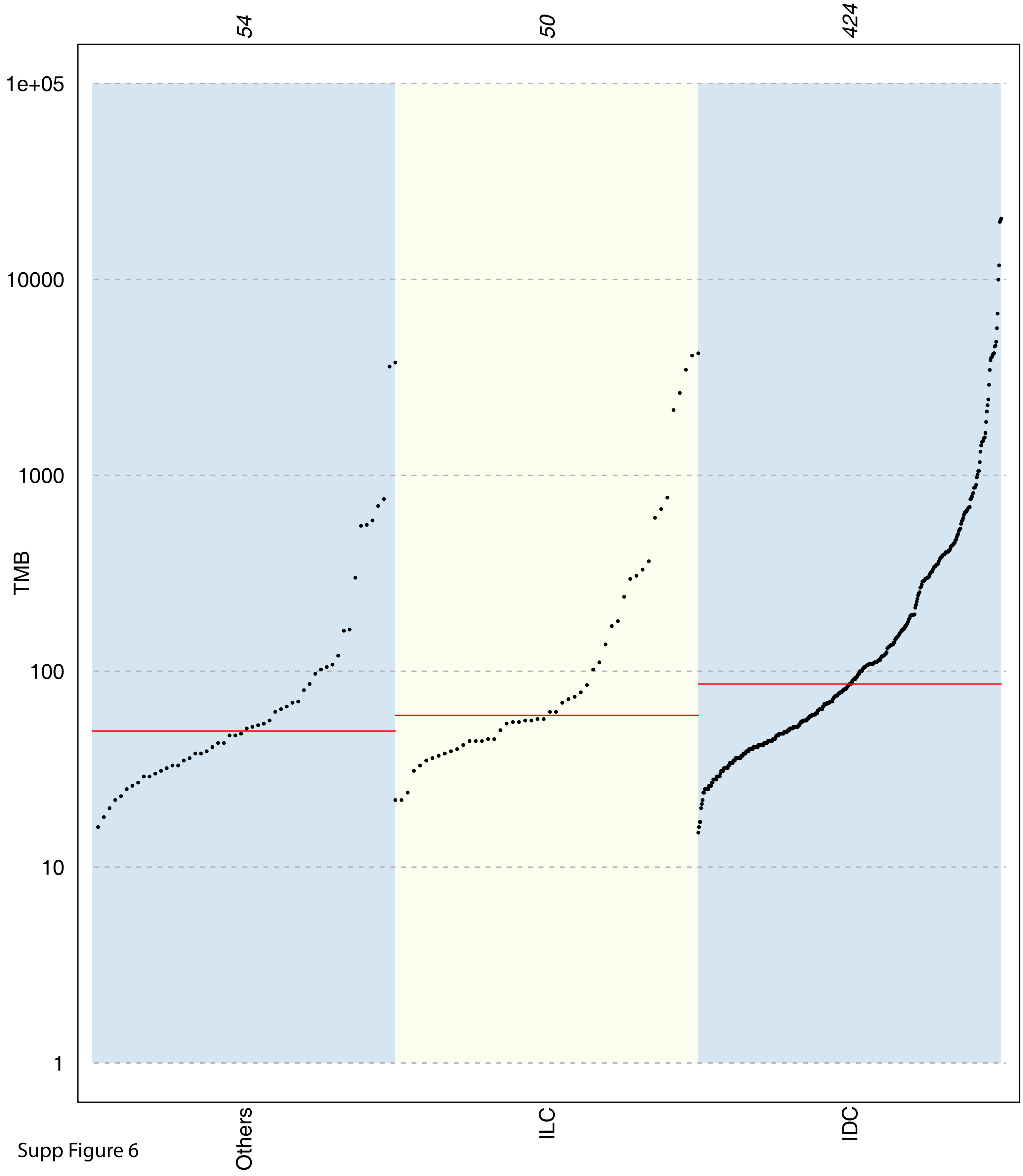


Supp Figure 6

Supplementary Figure 7

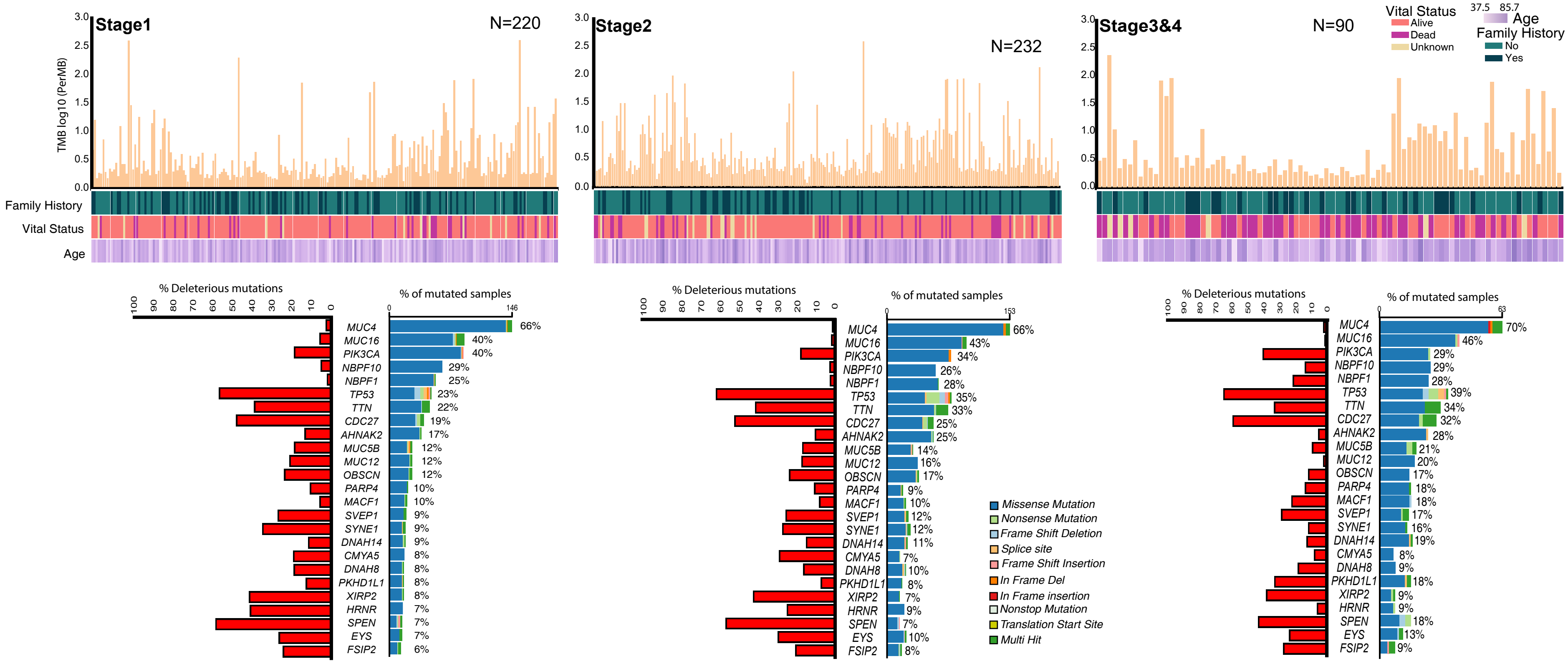

# Supplementary Figure 8

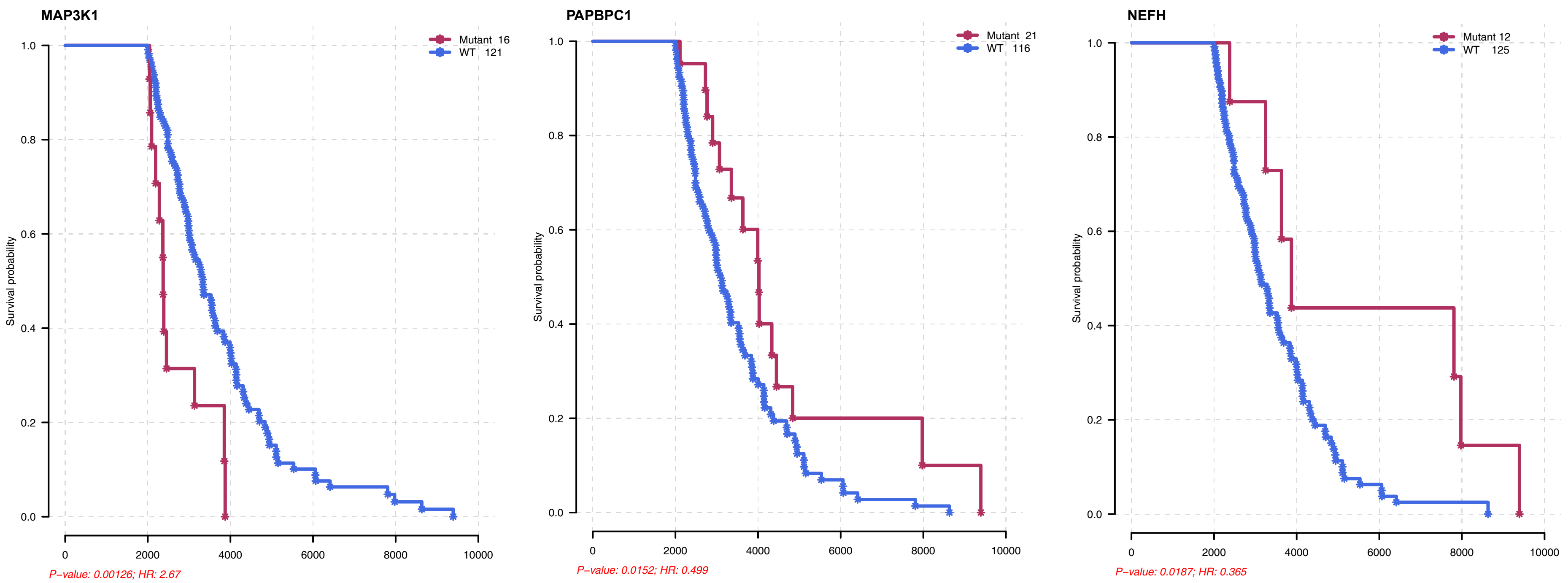

## Supplementary Figure 9

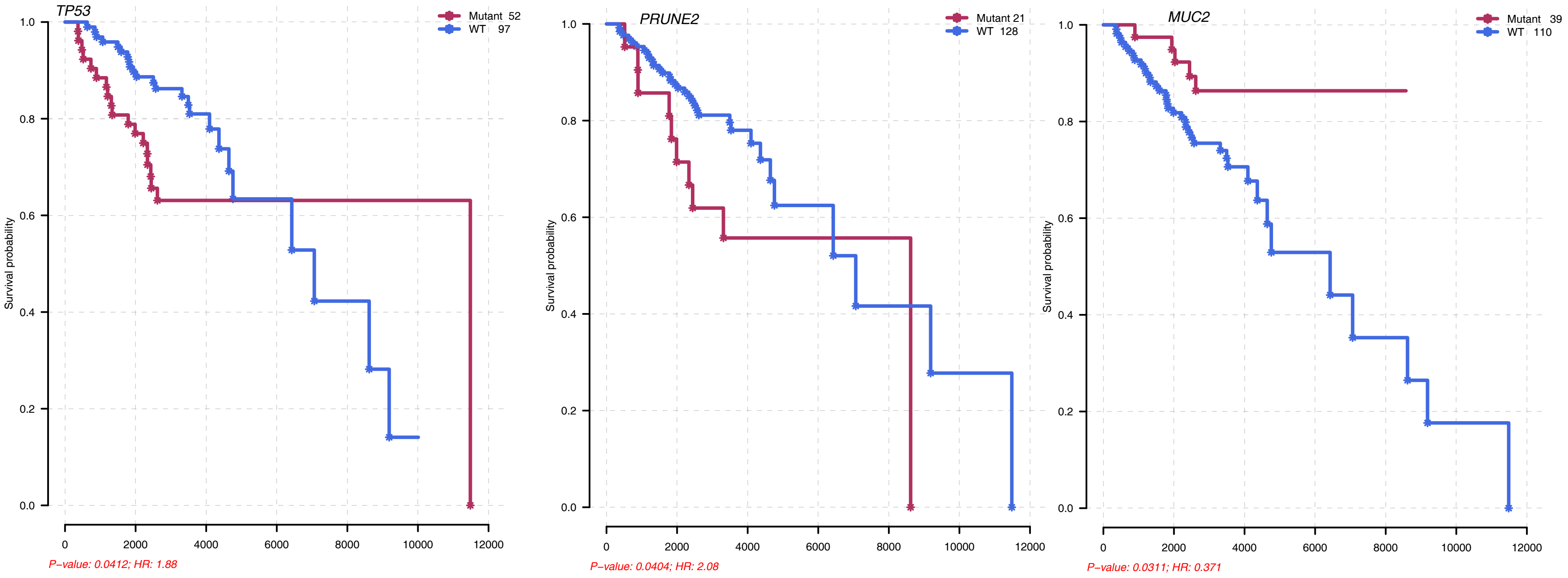

Supplementary Figure 10

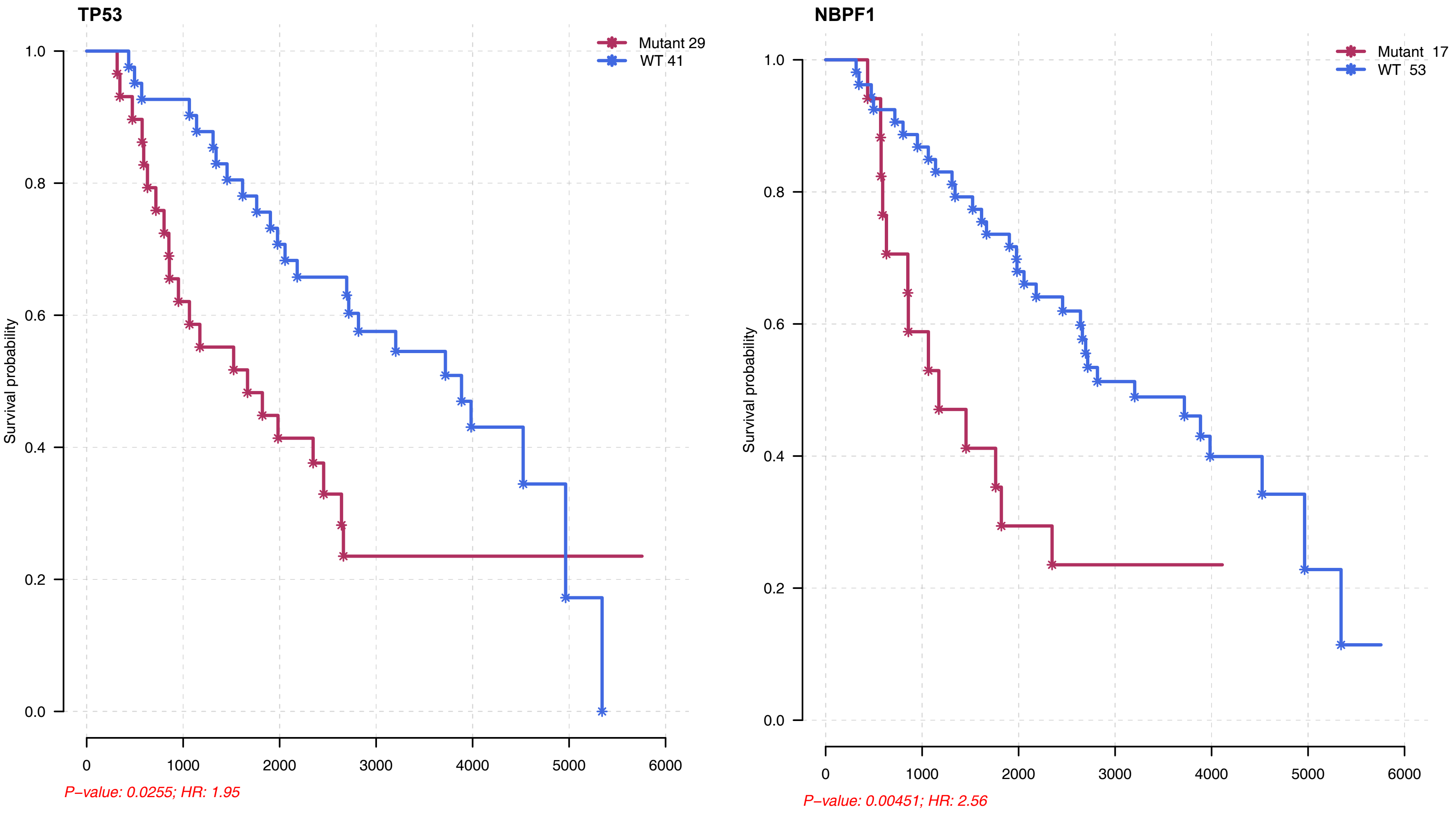

Supplementary Figure 11

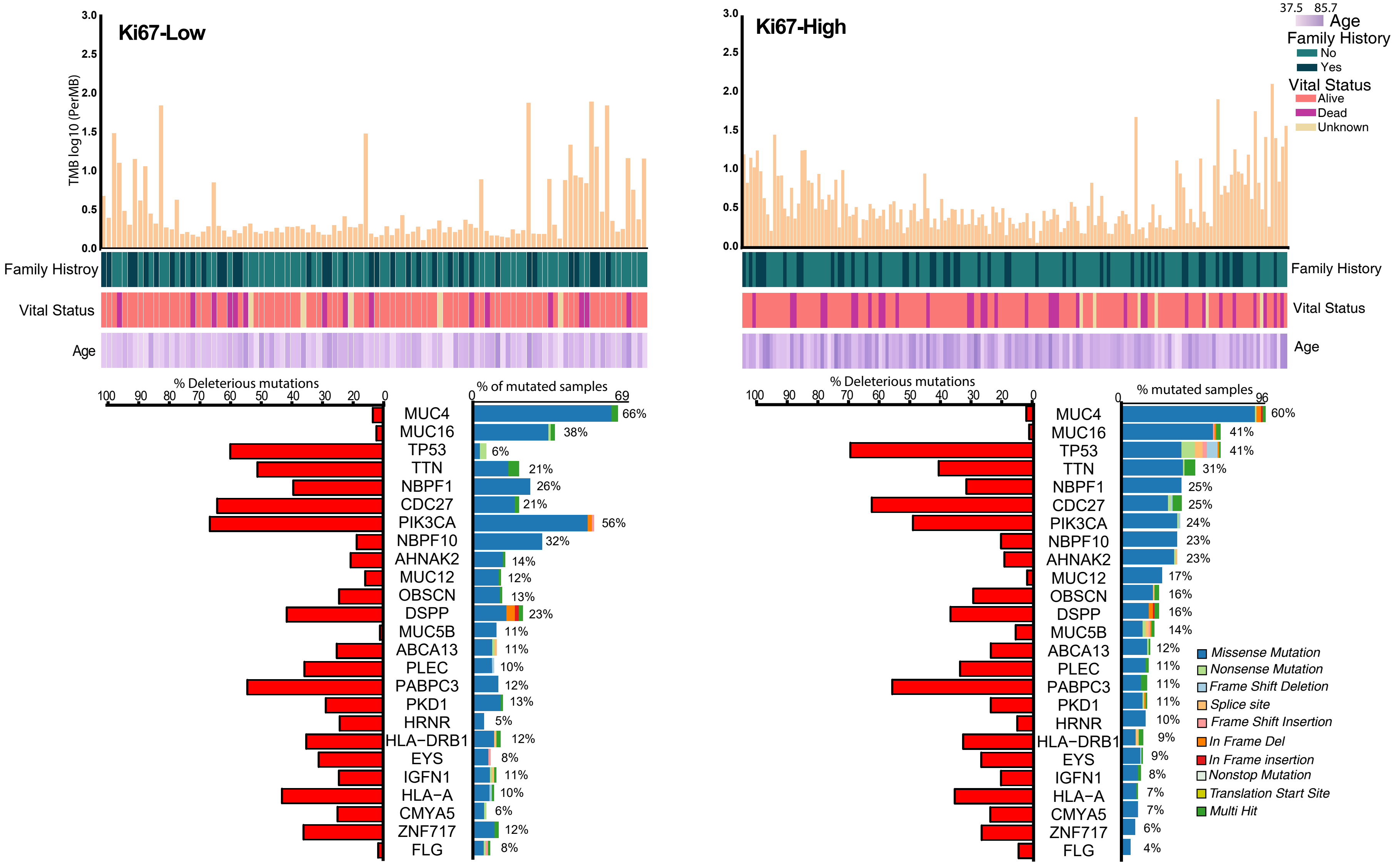

Supp Figure 12

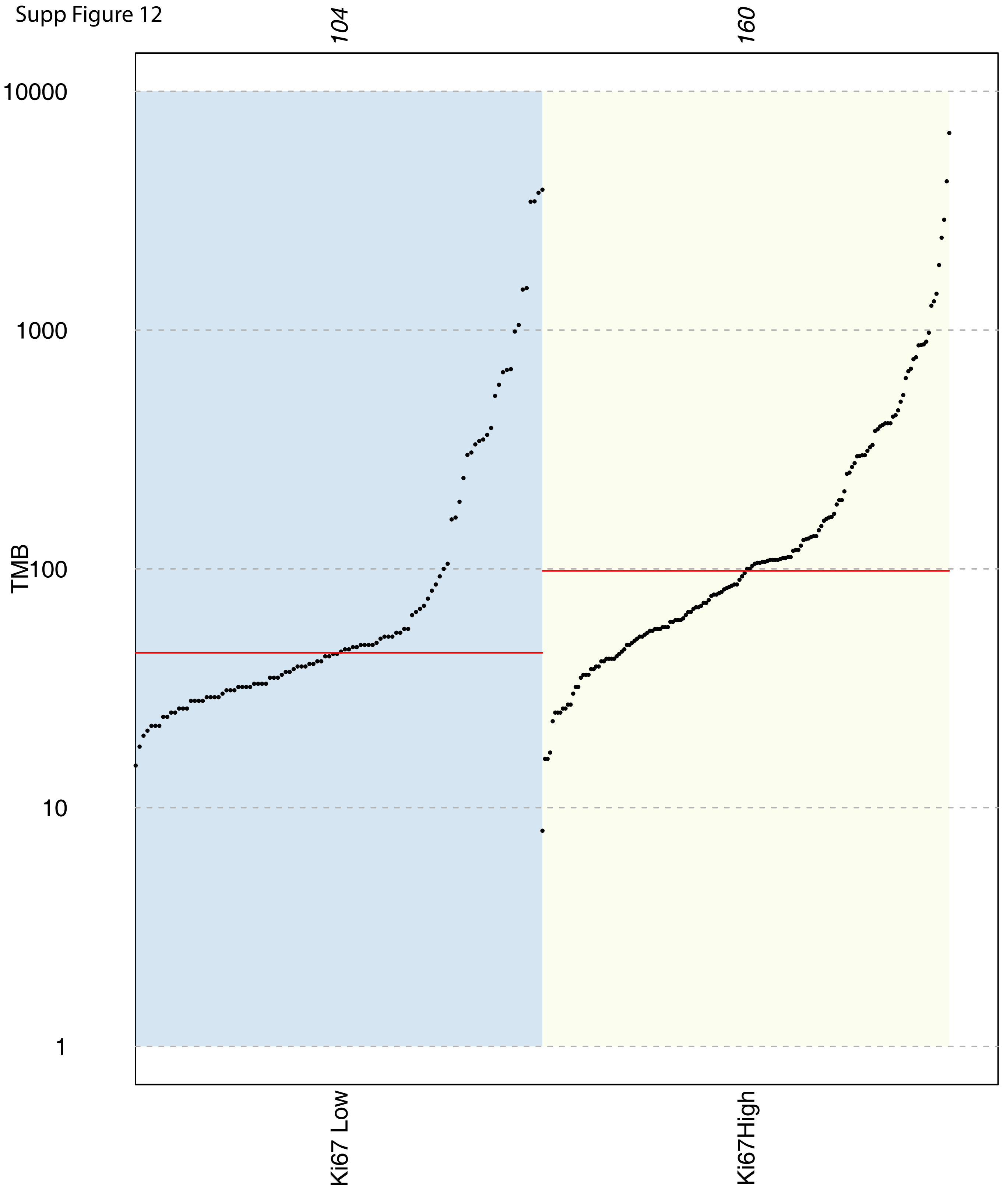

Supp Figure-13

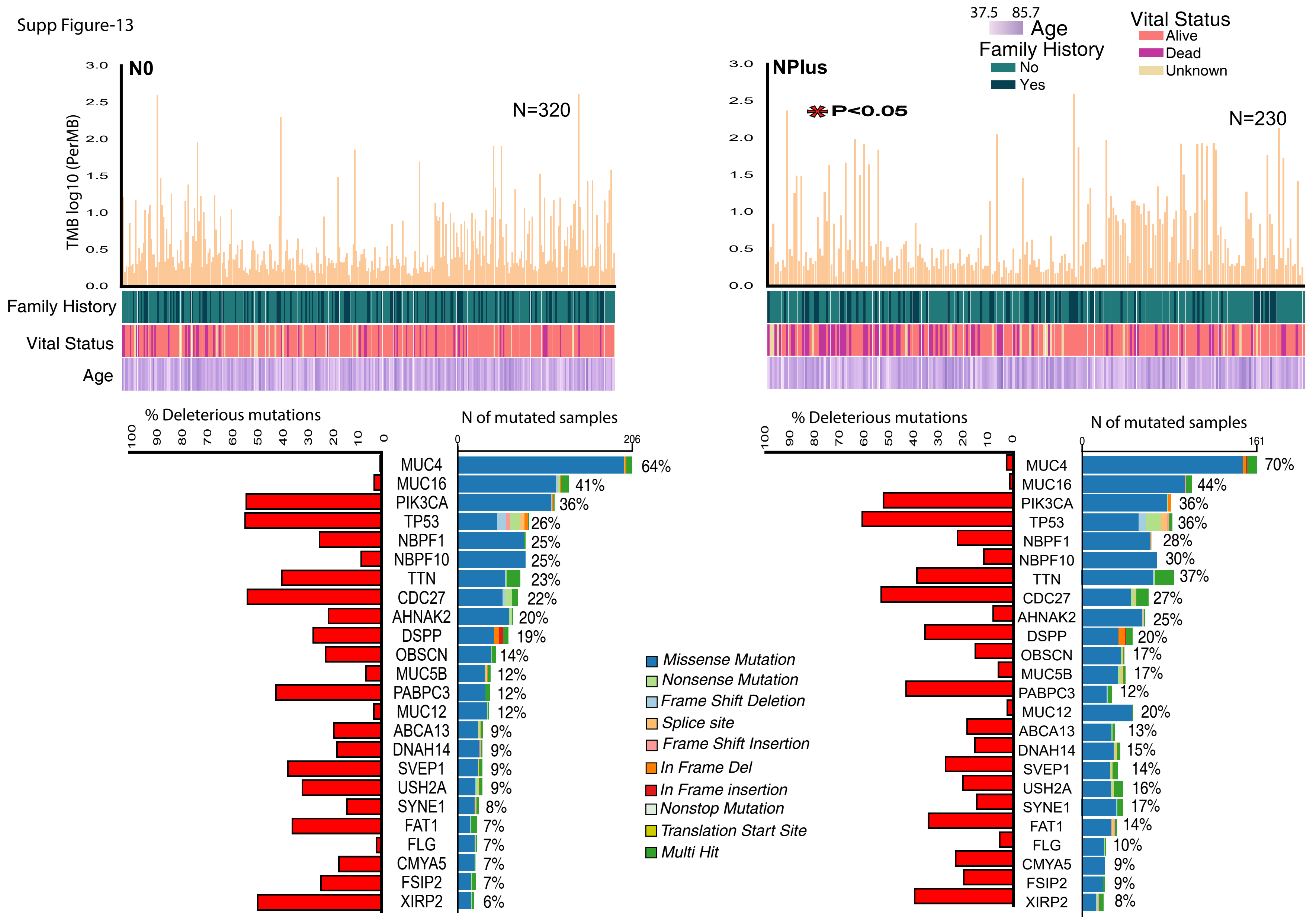

Supp Figure 14

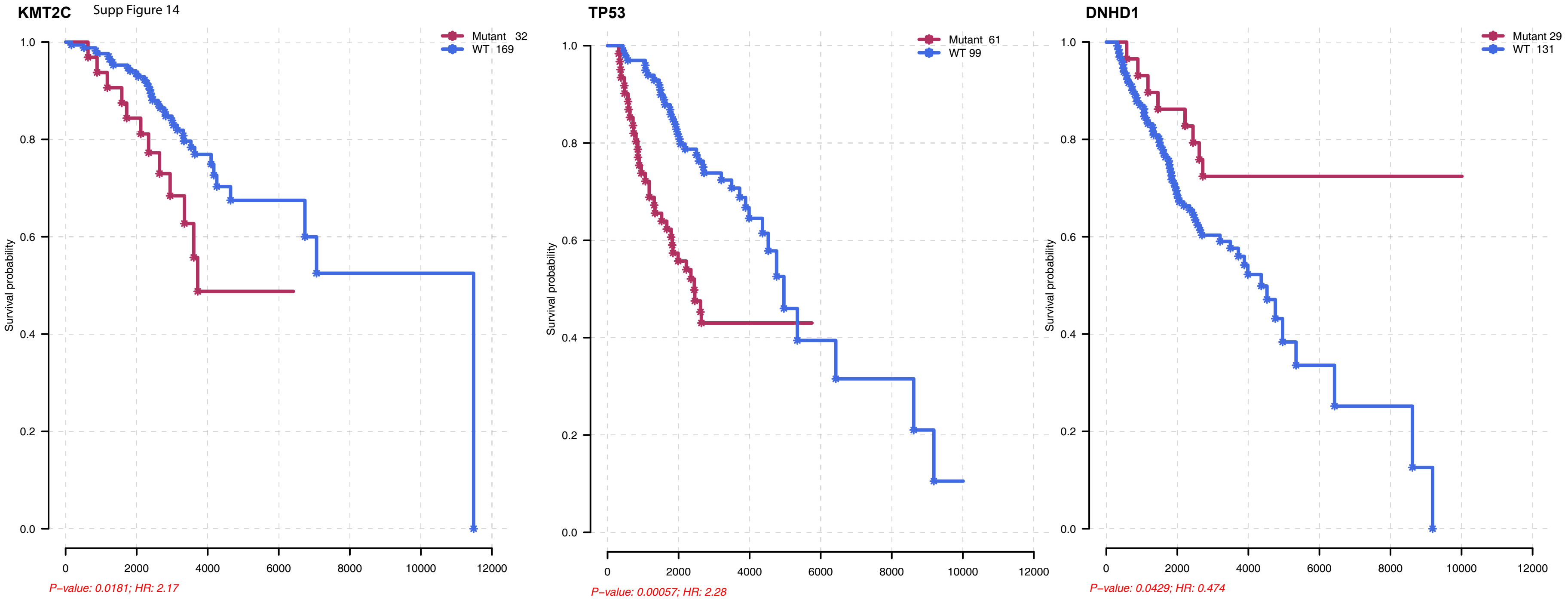

Supp Figure-15

| Gene | Subgroups | | | | | |
|---|---|---|---|---|---|---|
| AC011841.1 | ERPR-veHER2+ve ★ | Caucasians ★ | | | | |
| AHNAK2 | Grade2 | ERPR+veHER2-ve | AA | | | |
| HERC2 | Grade2 | IDC | | | | |
| MAP3K1 | Stage1 | Caucasians | | | | |
| MUC2 | Stage2 ★ | TN ★ | | | | |
| MUC5B | Grade2 | ERPR+veHER2-ve | Caucasians | | | |
| NBPF1 | Stage3&4 | Ki67-high | TN | AA | | |
| TP53 | Stage2 | Stage3&4 | Nplus | ERPR+veHER2-ve | IDC | Caucasians |
| PIK3CA | Grade1 | | | | | |
| CBFB | Grade1 | | | | | |
| PKD1 | Grade2 | | | | | |
| PABPC1 | Stage1 ★ | | | | | |
| NEFH | Stage1 ★ | | | | | |
| PRUNE2 | Stage2 | | | | | |
| KMT2C | N0 | | | | | |
| DNHD1 | Nplus ★ | | | | | |
| GATA3 | ERPR+veHER2-ve ★ | | | | | |
| GPR98 | ERPR+veHER2-ve | | | | | |
| NEB | ERPR+veHER2-ve | | | | | |
| NCOR2 | ILC | | | | | |
| SYNE1 | IDC | | | | | |
| NBPF10 | AA | | | | | |

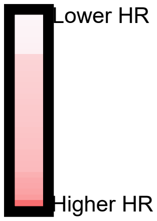

Relapse

| Gene | Subgroups | | |
|---|---|---|---|
| CRIPAK | Grade3 | | |
| NBPF1 | Grade3 | IDC | Caucasian |
| TP53 | Grade3 | IDC | Caucasian |
| RBMX | Grade3 | | |
| MACF1 | Grade3 ★ | | |
| MUC5B | Stage3&4 | | |
| HERC2 | IDC | | |
| NBPF10 | IDC | | |
| MUC12 | IDC | | |
| DNAH17 | Caucasian | | |

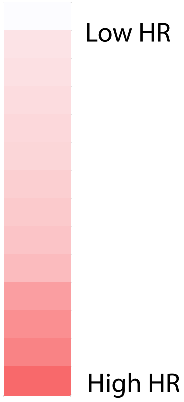


Metastasis

| Gene | Subgroups | | |
|---|---|---|---|
| AHNAK2 | Stage3&4 | Ki67-high ★ | |
| CRIPAK | Grade3 | Stage3&4 | N0 ★ |
| DNAH14 | Stage3&4 | IDC | |
| DSPP | ERPR+veHER2-ve | Caucasian | |
| GPR98 | Stage3&4 | ERPR+veHER2-ve | Caucasian |
| TP53 | IDC | Caucasian | |
| TTN | Stage3&4 | Caucasian | |
| MUC12 | N0 ★ | | |
| MUC4 | IDC | | |
| MUC5B | Caucasian | | |
| MUC6 | Ki67-high | | |
| NBPF1 | Grade3 | | |
| NBPF10 | IDC | | |
| OBSCN | Stage3&4 | | |
| PKHD1L1 | Stage3&4 | | |
| RYR3 | Stage3&4 | | |

Supp Figure-16
